# Supplementary material for: Changes over a decade in psychotropic prescribing for people with intellectual disabilities: prospective cohort study
Source: BMJ Open. 2020 Sep 10;10(9):e036862. doi: 10.1136/bmjopen-2020-036862 (PMC7488805; doi:10.1136/bmjopen-2020-036862)
Supplement: Supplementary data [file bmjopen-2020-036862supp001.pdf]

Supplementary table 1: Multivariable analysis of factors at T1 associated with psychotropic prescriptions at T2 for the linked cohort (N=545)

	**Antipsychotics**		**Antidepressants**		**Hypnotics/anxiolytics**		**Antiepileptics**		**Lithium***	
	**OR (95% CI)**	**p-value**	**OR (95% CI)**	**p-value**	**OR (95% CI)**	**p-value**	**OR (95% CI)**	**p-value**	**OR (95% CI)**	**p-value**
Male sex	0.96 (0.63, 1.47)	0.848	0.59 (0.38, 0.89)	0.013	0.83 (0.46, 1.50)	0.545	0.97 (0.66, 1.42)	0.874	0.92 (0.25, 1.09)	0.894
Age at T1	1.04 (1.02, 1.06)	<0.001	1.01 (1.00, 1.03)	0.167	1.00 (0.98, 1.02)	0.993	0.99 (0.98, 1.00)	0.158	1.04 (0.99, 1.09)	0.160
Level of ID	-	0.018	-	0.002	-	0.473	-	< 0.001	-	0.464
Mild	REF		REF		REF		REF		REF	
Moderate	1.71 (1.02, 2.87)	0.041	0.90 (0.55, 1.48)	0.680	1.16 (0.56, 2.38)	0.690	1.56 (0.99, 2.48)	0.057	2.91 (0.68, 12.53)	0.152
Severe	2.31 (1.29, 4.15)	0.005	0.57 (0.30, 1.08)	0.083	0.75 (0.28, 1.97)	0.554	1.66 (0.96, 2.86)	0.071	0.90 (0.09, 8.80)	0.928
Profound	1.07 (0.53, 2.18)	0.853	0.22 (0.09, 0.56)	0.001	1.71 (0.71, 4.11)	0.229	3.57 (1.96, 6.5)	0.001	1.39 (0.14, 13.74)	0.780
Mental ill-health**	3.50 (2.02, 6.06)	< 0.001	2.73 (1.58, 4.70)	< 0.001	2.79 (1.34, 5.81)	0.006	1.33 (0.78, 2.27)	0.294	-	-
Problem behaviours	5.47 (3.25, 9.22)	< 0.001	2.75 (1.59, 4.76)	< 0.001	2.08 (1.00, 4.34)	0.050	1.35 (0.83, 2.17)	0.225	-	-

*Mental illness and problem behaviours excluded from Lithium model due to small numbers **Not including problem behaviours
