# Supplementary material for: Changes over a decade in psychotropic prescribing for people with intellectual disabilities: prospective cohort study
Source: BMJ Open. 2020 Sep 10;10(9):e036862. doi: 10.1136/bmjopen-2020-036862 (PMC7488805; doi:10.1136/bmjopen-2020-036862)
Supplement: Author's manuscript [file bmjopen-2020-036862.draft_revisions.pdf]

BMJ Open is committed to open peer review. As part of this commitment we make the peer review history of every article we publish publicly available.

When an article is published we post the peer reviewers' comments and the authors' responses online. We also post the versions of the paper that were used during peer review. These are the versions that the peer review comments apply to.

The versions of the paper that follow are the versions that were submitted during the peer review process. They are not the versions of record or the final published versions. They should not be cited or distributed as the published version of this manuscript.

BMJ Open is an open access journal and the full, final, typeset and author-corrected version of record of the manuscript is available on our site with no access controls, subscription charges or pay-per-view fees (http://bmjopen.bmj.com).

If you have any questions on BMJ Open's open peer review process please email info.bmjopen@bmj.com

BMJ Open

Changes over a decade in psychotropic prescribing for people with intellectual disabilities: prospective cohort study

Journal:	*BMJ Open*
Manuscript ID	bmjopen-2020-036862
Article Type:	Original research
Date Submitted by the Author:	07-Jan-2020
Complete List of Authors:	Henderson, Angela; University of Glasgow, Institute of Health and Wellbeing<br>Mcskimming, Paula; University of Glasgow, Robertson Centre for Biostatistics<br>Kinnear, Deborah; University of Glasgow, Institute of Health and Wellbeing<br>McCowan, Colin ; University of Saint Andrews, School of Medicine<br>Allan, Linda; University of Glasgow, Institute of Health and Wellbeing<br>Cooper, Sally-Ann; Glasgow University , Institute of Health and Wellbeing
Keywords:	MENTAL HEALTH, PSYCHIATRY, CLINICAL PHARMACOLOGY

SCHOLARONE™ Manuscripts

I, the Submitting Author has the right to grant and does grant on behalf of all authors of the Work (as defined in the below author licence), an exclusive licence and/or a non-exclusive licence for contributions from authors who are: i) UK Crown employees; ii) where BMJ has agreed a CC-BY licence shall apply, and/or iii) in accordance with the terms applicable for US Federal Government officers or employees acting as part of their official duties; on a worldwide, perpetual, irrevocable, royalty-free basis to BMJ Publishing Group Ltd ("BMJ") its licensees and where the relevant Journal is co-owned by BMJ to the co-owners of the Journal, to publish the Work in this journal and any other BMJ products and to exploit all rights, as set out in our licence.

The Submitting Author accepts and understands that any supply made under these terms is made by BMJ to the Submitting Author unless you are acting as an employee on behalf of your employer or a postgraduate student of an affiliated institution which is paying any applicable article publishing charge ("APC") for Open Access articles. Where the Submitting Author wishes to make the Work available on an Open Access basis (and intends to pay the relevant APC), the terms of reuse of such Open Access shall be governed by a Creative Commons licence – details of these licences and which Creative Commons licence will apply to this Work are set out in our licence referred to above.

Other than as permitted in any relevant BMJ Author's Self Archiving Policies, I confirm this Work has not been accepted for publication elsewhere, is not being considered for publication elsewhere and does not duplicate material already published. I confirm all authors consent to publication of this Work and authorise the granting of this licence.

For peer review only

Title: Changes over a decade in psychotropic prescribing for people with intellectual disabilities: prospective cohort study

Authors: Angela Henderson[1], Deputy Director, Scottish Learning Disabilities Observatory
Paula McSkimming[2], Biostatistician
Deborah Kinnear[1], Senior Lecturer
Colin McCowan[3], Professor in Health Data Science
Linda Allan[1], Honorary Clinical Associate Professor
*Sally-Ann Cooper[1], Professor of Learning Disabilities

*Correspondence. Sally-Ann.Cooper@Glasgow.ac.uk 0141 211 0690

1. Institute of Health and Wellbeing, University of Glasgow, Mental Health and Wellbeing research group, 1st Floor, Administrative Building, Gartnavel Royal Hospital, 1055 Great Western Road, Glasgow, G12 0XH
2. Robertson Centre for Biostatistics, Institute of Health and Wellbeing, University of Glasgow, Boyd Orr Building, University Avenue, Glasgow, G12 8QQ
3. School of Medicine, University of St Andrews, North Haugh, St Andrews KY16 9TF

Word count: 3235

For peer review only - http://bmjopen.bmj.com/site/about/guidelines.xhtml

ABSTRACT

OBJECTIVES: To investigate changes in psychotropic prescribing in the intellectual disabilities population over a 10 year period, and associated mental ill-health diagnoses.

DESIGN: (a) Comparison of cross-sectional data in 2002-2004 (T1) and 2014 (T2). (b) Longitudinal cohort study with detailed health assessments at T1, and record linkage to prescribing data in T2.

SETTING: General community.

PARTICIPANTS: (a) 1,190 adults with intellectual disabilities in T1 compared with 3,906 adults with intellectual disabilities in T2. (b) 545/1,190 adults with intellectual disabilities in T1 were alive and their records linked to T2 prescribing data.

MAIN OUTCOME MEASURES: Psychotropics prescribed.

RESULTS: (a) 50.7% (603/1,190) in T1, and 48.2% (1,881/3,906) in T2 were prescribed 1+ psychotropics: antipsychotics 24.5% (292/1,190) in T1 and 16.7% (653/3,906) in T2, antidepressants 11.2% (133/1,190) in T1 and 16.7% (653/3,906) in T2. 30.0% (62/292) prescribed antipsychotics in T1 had psychosis or bipolar disorder, 33.2% (97/292) had no mental ill-health or problem behaviours, 20.6% (60/292) had problem behaviours but no psychosis/bipolar disorder. (b) Psychotropics increased from 47.0% (256/545) in T1 to 57.8% (315/545) in T2 ($p<0.238$): antipsychotics did not change (OR=1.18; CI (0.87, 1.60); $p=0.280$), there was an increase for antidepressants (OR=2.80; CI 1.96, 4.00; $p<0.001$), hypnotics/anxiolytics (OR=2.19; CI 1.34, 3.61; $p=0.002$), and antiepileptics (OR=1.40; CI 1.06, 1.84; $p=0.017$). Antipsychotic prescribing increased for people with problem behaviours in T1 (OR=6.45, CI 4.41, 9.45; $p<0.001$), more so than for people with other mental ill-health in T1 (OR=4.10, CI 2.75, 6.11; $p<0.001$).

CONCLUSIONS – Despite concerns about antipsychotic prescribing and guidelines recommending their withdrawal, it appears that whilst fewer antipsychotic prescriptions were initiated by T2 than in T1, people were not withdrawn from them once commenced. People with problem behaviours had increased rates of prescribing. There was also a striking increase in antidepressant prescriptions. Adults with intellectual disabilities need frequent and careful medication reviews.

Key words: intellectual disabilities, psychotropics, antipsychotics, antidepressants, hypnotics, anxiolytics, anti-epileptics, lithium, mental ill-health

Article summary

For peer review only - http://bmjopen.bmj.com/site/about/guidelines.xhtml

Strengths and limitations

The strengths of this study are:

- The large cohort size, longitudinal design, detailed ascertainment of the population with intellectual disabilities, and the detailed health assessments at T1.
- The whole cohorts were population-based at T1 and T2, an representative of the population with intellectual disabilities, the linked cohort was similar in characteristics with the whole cohort at T1 suggesting it is also representative and therefore that the results are generalisable.

The limitations of this study are:

- Only 73% of general practices agreed to data extraction, and this combined with deaths are likely to be the main reasons for 545/1,190 of the participants being linked in the T2 data, 10 years later.
- The different methods of data collection, with specialist individual assessments at T1 and electronic data extraction at T2; in particular there is a large proportion of missing information and may be inaccuracies on recorded level of intellectual disabilities in the general practitioner data at T2, so comparison of this variable between the T1 and T2 cohorts is limited.

The study did not investigate changes in dosages, polypharmacy or duration of use and there is lack of mental ill-health data at T2.

INTRODUCTION

Mental ill-health is common in people with intellectual disabilities. (1) Their prevalence of psychosis is reported to be around 4% based on cross-sectional data and the rate of people with a first psychotic episode is about 10 times that of the general population. (2) However, despite these relative high rates of psychosis, antipsychotics are often prescribed for people with intellectual disabilities who do not have a record of severe mental ill-health (3, 4), often for problem behaviours, (5-9) despite limited evidence to support their use beyond short-term sedation. (7) Indeed, 71% of people with intellectual disabilities prescribed antipsychotics have been reported to not have a record of serious mental ill-health. (10) This is important, as antipsychotics have numerous unpleasant, disabling, painful, and disfiguring side effects, some of which are life threatening such as tardive dyskinesia, cardiac arrhythmias, and sudden cardiac death. (11-13)

For peer review only - http://bmjopen.bmj.com/site/about/guidelines.xhtml

Concerns have repeatedly been raised about this overuse of antipsychotics, and the need for more proportionate prescribing. (7, 14-16) NHS England launched a national campaign: “Stopping over medication of people with a learning disability, autism or both (STOMP)” in partnership initially with the Royal College of General Practitioners, Royal College of Psychiatrists, Royal College of Nursing, Royal Pharmaceutical Society, and British Psychological Society, and subsequently additional partners. Guidelines from STOMP, the National Institute for Health and Care Excellence, and the Royal College of Psychiatrists highlight that prescribers, where appropriate, should reduce or withdraw antipsychotics for people with intellectual disabilities who do not have psychosis. (7, 17, 18) However, there is very little empirical evidence from the UK on any changes in antipsychotic prescribing over time. An exception is a study that extracted data from general practice records on 33,016 adults with a record of intellectual disabilities, with a median follow-up of 5.5 years. (10) They reported the incidence of a new psychotropic prescription to be 518/10,000 person years. Prescription of antipsychotics fell by 4% per year over the study period, as did mood stabilisers, whilst there was no consistent trend for antidepressants or anxiolytics/hypnotics. They reported that 47% with “challenging behaviour” had received antipsychotic drugs, and only 12% had a record of severe mental ill-health, and that 26% prescribed antipsychotics did not have a record of severe mental ill-health or “challenging behaviour”. The study was limited by “challenging behaviour” being identified from a heterogeneous list of 45 Read codes, due to the limitations of the Read coding system for problem behaviours, combined with incomplete and variable recording practices which do not always accurately reflect a person’s health. (10) Another study from Australia investigated psychotropic medication use between 1999 and 2015 in a cohort of 138 participants (19) and also found a strong association between problem behviours and psychotropic medication. However, in this cohort the study reported that once psychotropic medications were prescribed they were unlikely to be removed, and observed little change in prescribing of antipsychotics between 1999 and 2015 (24/138 (24%) to 23/92 (23%)). There was also a sharp increase in the prescribing of antidepressants from 16.7% to 36.1% across the same period. However whilst this was a longitudinal cohort not all participants took part in all waves of data collection, therefore it is not possible to ascertain within group changes in prescribing.

For peer review only - http://bmjopen.bmj.com/site/about/guidelines.xhtml

The aim of this study is to investigate changes over a decade in psychotropic prescribing for adults with intellectual disabilities, and the diagnoses associated with antipsychotics, from detailed psychiatric assessments.

METHODS

Ethical approval

NHS Greater Glasgow Primary Care Trust - Community & Mental Health Research Ethics Committee granted ethical approval (project number 01/44). Between 2002-2004 (T1), individual consent to participate was taken in line with Scottish law. In 2014 (T2), 191/263 (73%) general practices in NHS Greater Glasgow and Clyde area participated, and the NHS Greater Glasgow and Clyde Local Privacy Advisory Committee approved electronic extraction and linkage of primary care records.

Participants

Adults with intellectual disabilities, aged ≥16 years, living in part of the NHS Greater Glasgow area were identified through social work services for people with intellectual disabilities; local authority funding arrangements for persons receiving paid support of any kind, including day opportunities; local specialist health services for people with intellectual disabilities; the Health Board; and general practices. 1,202 participants were recruited to a longitudinal study between 2002 and 2004 (T1), and had detailed health assessments at that time. 1,190/1,202 were aged ≥18 years. In 2014 (T2), data was extracted from primary care records on participants aged ≥14 years in 73% of general practices with Read coding of being on the intellectual disabilities register (N=4,066). 3,906 were aged ≥18 years. The intellectual disabilities register was established between 2000-2001 with joint work between all general practices in the area, and the intellectual disabilities primary care liaison team. A check was made by community intellectual disabilities nurses of each person on the register to ensure they did indeed have intellectual disabilities, and those that did not were removed from it. The register was then annually updated.

Process and measures

Semi-structured individual health assessments, including medication review, assessment of level of intellectual disabilities, mental ill-health symptoms including problem behaviours and autism, were conducted at T1 by one of six intellectual disabilities nurses and one of

For peer review only - http://bmjopen.bmj.com/site/about/guidelines.xhtml

three general practitioners with special interest in intellectual disabilities. The 54% of individuals identified with possible, probable, or definite mental ill-health (including problem behaviours and autism) were then assessed by the study's psychiatrists who were specialists in intellectual disabilities psychiatry. Information from each person's psychiatric assessment was reviewed by two psychiatrists who agreed the classification of the mental ill-health. Drugs were coded using British National Formulary (BNF) codes. Details have been previously reported. (1)

At T2, the 4,066 adults with intellectual disabilities identified from primary care records were record linked to Prescribing Information System (PIS) data, using the Community Health Index (CHI), which is the NHS patient identifier number, unique to each person. PIS is Scotland's electronic record of all encashed prescriptions, and includes a record of the BNF code of each prescribed drug.(20) Prescribing information was then extracted by BNF codes for the 4,066 adults with intellectual disabilities to identify all prescriptions of antipsychotics, antidepressants, antiepileptics, lithium, and hypnotics/anxiolytics across a 12-week prescribing window in 2014. Next, again using the CHI, the T1 participant were identified in the T2 data, so their prescriptions could be compared across the decade. Only participants with compete data who were aged 18 years and over were included in the analyses (supplementary figure).

For peer review only

Statistical analysis

Subject characteristics and prescribing information were summarised descriptively with mean and standard deviation (SD) for continuous outcomes and number and percentage for categorical outcomes at each time point (T1 and T2). To investigate psychotropic medication prescribing patterns over the two time points in the study, McNemar's tests were carried out with the linked cohort for whom there were records at each of T1 and T2. This analysis was extended to explore if subject characteristics at T1 had an association with a change in prescription outcomes over time using multivariable repeated measures logistic regression models. Time was fitted along with sex, age, level of intellectual disabilities, having mental ill-health (excluding problem behaviours) and having problem behaviours. Multivariable logistic regression models were also fitted with the above T1 subject characteristics to explore their association with prescribing outcomes at T2 only. Odd ratios are reportedwith corresponding 95% confidence intervals (CIs) and p-values. A p-value of less than 0.05 is

For peer review only - http://bmjopen.bmj.com/site/about/guidelines.xhtml

considered as statistically significant. Statistical analyses were conducted using SAS version 9.3.

RESULTS

Participant characteristics of the whole cohorts

Data for those who had incomplete data at T1 and for those who were aged under 18 years at either time point were excluded from further analyses. Table 1 shows age, sex, level of intellectual disabilities and psychotropic prescribing at T1 (n=1,190) and T2 (n=3,906), and mental ill-health diagnoses at T1. No mental health data were available at T2.

Table 1. Participant characteristics for the whole cohorts at T1 and T2

Characteristic	T1 aged 18+years (N=1,190)	T2 aged 18+ years (N=3,906)
**Age Mean (SD)**	44.6 (14.3)	45.4 (15.5)
**Sex N(%):**		
Male	671 (56.4)	2,260 (57.9)
Female	519 (43.6)	1,646 (42.1)
**Level of intellectual disabilities N(%):**		
Mild	451 (37.9)	1047 (26.8)
Moderate	319 (26.8)	859 (22.0)
Severe	233 (19.6)	595 (15.2)
Profound	187 (15.7)	197 (5.0)
Unknown	0	1,208 (30.9)
**Type of mental ill-health N(%):**		
Psychosis, including psychosis in remission	52 (4.4)	Not collected
Problem behaviours	244 (20.5)	
Autism	80 (6.7)	
ADHD	15 (1.3)	
Unipolar depression	51 (4.3)	
Bipolar disorder	21 (1.8)	
Anxiety disorders	32 (2.7)	
Organic disorder	20 (1.7)	
Personality disorder	9 (0.8)	
Obsessive compulsive disorder	7 (0.6)	
Psychosexual disorder	< 5	
Other	15 (1.3)	
Mental ill-health (including problem behaviours)	438 (36.8)	
Mental ill-health (excluding problem behaviours)	194 (16.3)	

Prescribing for the whole cohorts

For peer review only - http://bmjopen.bmj.com/site/about/guidelines.xhtml

For peer review only

At least one psychotropic was prescribed at T1 for 50.7% (603/1,190), and at T2 for 48.2% (1,881/3,906) (table 2). At T1, antipsychotics were prescribed for 24.5% (292/1,190), and at T2 for 16.7% (653/3,906). At T1, antidepressants were prescribed for 11.2% (133/1,190), and at T2 for 19.1% (746/3,906). There were similar prescribing rates at T1 and T2 for hypnotics/anxiolytics, lithium, and anti-epileptics.

The types of mental ill-health experienced by the 292 participants at T1 who were taking antipsychotics are shown in table 3. The most common diagnosis within this group was problem behaviours at 40.1% (119/292). Of note, 33.2% (97/292) of the adults taking antipsychotics did not have any identified mental ill-health nor problem behaviours. Figure 1 demonstrates the overlap between groups of the people who were taking antipsychotics at T1 and selected diagnoses.

Figure 1. Types of mental ill-health experienced by adults prescribed antipsychotics at T1 n=292

Table 3 also shows the types of mental ill-health experienced by the 230 participants at T1 who were taking antipsychotics, after excluding people with psychosis (or psychosis in remission) or bipolar disorder (given that they would be expected to be prescribed antipsychotics, and given the considerable overlap between disorders shown in Figure 1). Most strikingly, 97/230 (42.2%) on antipsychotics had no mental ill health or problem behaviours. The proportion of people in each diagnostic category and without co-occurring psychosis or bipolar disorder who were taking antipsychotics was considerable for all types: 11.7% (27/230) for autism, 7.0% (16/230) for unipolar depression, 2.6% (6/229) for anxiety disorders and 2.2% (5/230) or less for all other diagnoses.

Table 2 Psychotropics prescribed for the whole cohorts at T1 and T2

**Prescriptions**	**T1 aged 18+ years (N=1,190)**	**T2 aged 18+ years (N=3, 906)**
Any psychotropic drug	603 (50.7)	1,881 (48.2)
Antipsychotics	292 (24.5)	653 (16.7)
Antidepressants	133 (11.2)	746 (19.1)
Antiepileptics	333 (28.0)	1,028 (26.3)
Lithium	14 (1.2)	31 (0.8)
Hypnotics/anxiolytics	81 (6.8)	305 (7.8)

For peer review only - http://bmjopen.bmj.com/site/about/guidelines.xhtml

Missing data	0	3 (0.1)

Table 3. Types of mental ill-health at T1 experienced by adults prescribed antipsychotics at T1, and after excluding people with psychosis and bipolar disorder

Mental ill-health at T1		Adults (18+ years) taking antipsychotics at T1	
**Diagnosis**	**All (N=1,190)**	**All (N=292)**	**Excluding people with psychosis and bipolar disorder (N=230)**
Psychosis, including psychosis in remission	52	45 (15.4)	-
Problem behaviours	244	119 (40.8)	100 (43.5)
Autism	80	30 (10.3)	27 (11.7)
ADHD	15	12 (4.1)	11 (4.8)
Unipolar depression	51	20 (6.9)	16 (7.0)
Bipolar disorder	21	17(5.8)	-
Anxiety disorders	32	7 (2.4)	6 (2.6)
Organic disorder	20	5 (1.7)	< 5
Personality disorder	9	5 (1.7)	< 5
Obsessive Compulsive Disorder	7	3 (1.0)	< 5
Psychosexual disorder	< 5	< 5	< 5
Other	15	6 (2.1)	5 (2.2)
Mental ill-health (including problem behaviours)	438	195 (66.8)	133 (57.8)
Mental ill-health (excluding problem behaviours)	194	76 (26.3)	33 (14.4)
No mental ill-health nor problem behaviours	752	97 (33.2)	97 (42.24)

- **Insert figure 1 here. Types of mental ill-health experienced by adults prescribed antipsychotics at T1 -**

Participant characteristics of the linked cohort

For peer review only - http://bmjopen.bmj.com/site/about/guidelines.xhtml

The linked cohort included the 545 adults who were both in the T1 cohort and identified within the GP records at T2. Table 4 shows their age, sex, level of intellectual disabilities, and mental ill-health at T1. They appear to be broadly representative of the whole cohort at T1 on these characteristics.

Table 4. Participant characteristics at T1 for people in the linked cohort

Characteristic	T1 (aged 18+ years) N=545
**Age Mean (SD)**	41.8 (13.2)
**Sex N (%):**	
Male	322 (59.1)
Female	223 (40.9)
**Level of intellectual disabilities N (%):**	
Mild	237 (43.5)
Moderate	154 (28.3)
Severe	89 (16.3)
Profound	65 (11.9)
Missing	0
**Type of mental ill-health N (%):**	
Psychosis, including psychosis in remission	32 (5.9)
Problem behaviours	109 (20.0)
Autism	38 (7.0)
ADHD	8 (1.5)
Unipolar depression	23 (4.2)
Bipolar disorder	10 (1.8)
Anxiety disorders	14 (2.6)
Organic disorder	< 5
Personality disorder	7 (1.3)
Obsessive compulsive disorder	< 5
Psychosexual disorder	<5
Other	6 (1.1)
Mental ill-health (including problem behaviours)	190 (34.9)
Mental ill-health (excluding problem behaviours)	81 (14.9)

Prescribing for the linked cohort

At least one psychotropic medication was prescribed at T1 for 47.0% (256/545), and for 57.8% (315/545) at T2 (table 5) which is a significant increase over time ($p<0.001$). Antidepressants were prescribed for 9.9% (54/545) at T1, and at T2 for 22.0% (120/545), showing a significant increase ($p<0.001$). At T1, hypnotics/anxiolytics were prescribed for 4.6% (25/545), and at T2 for 9.4% (51/545), a significant increase ($p<0.001$). At T1,

For peer review only - http://bmjopen.bmj.com/site/about/guidelines.xhtml

antiepileptics were prescribed for 24.8% (128/545), and at T2 for 31.0% (169/545), a significant increase (p<0.001). There were similar prescribing rates at T1 and T2 for antipsychotics and lithium.

Table 5: Psychotropic medications prescribed for the linked cohort at T1 and T2

Medication group	T1 (N=545)	T2 (545)	P-value
**Any psychotropic medication**	256 (47.0%)	315 (57.8%)	p<0.001
**Antipsychotics**	128 (23.5%)	142 (26.1%)	p=0.099
**Antidepressants**	54 (9.9%)	120 (22.0%)	p<0.001
**Hypnotics/anxiolytics**	25 (4.6%)	51 (9.4%)	P<0.001
**Antiepileptics**	135 (24.8%)	169 (31.0%)	P<0.001
**Lithium**	7 (1.3%)	10 (1.8%)	P=0.180

The multivariable repeated measures regression analyses, adjusting for sex, age, level of intellectual disabilities, having mental ill-health (excluding problem behaviours) and having problem behaviours at T1 , (Table 6) shows no change in antipsychotic prescribing in the linked cohort over the decade (OR = 1.18; CI 0.87 to 1.60; p = 0.280), an increase in antidepressants (OR=2.80; CI 1.95 to 4.00; p<0.001), hypnotics/anxiolytics (OR=2.19; CI 1.33 to 3.61; p=0.002), and antiepileptic prescribing (OR=1.40; CI 1.06 to 1.84; p = 0.017). Sex was not independently associated with a change in prescribing, except that women were more likely to have an increase in antidepressants than men over time (OR = 0.53, CI 0.37 to 0.78; p<0.001). Older age had a small effect for increased prescribing rates for all classes of drugs except hypnotics and anxiolytics over time. Similarly effects are seen for the level of intellectual disabilities, but it was only a linear gradient for antiepileptics (increased prescribing with severity of intellectual disabilities) and antidepressants (increased prescribing at lower levels of intellectual disabilities) and did not effect lithium prescribing. As expected, participants with a diagnosed mental health problem (excluding problem behaviours) at T1 were more likely to have increased prescribing of antipsychotics (OR=4.11, CI 2.76 to 6.11; p < 0.001), antidepressants (OR=3.90, CI 2.53 to 6.02; p < 0.001), and hypnotics/anxiolytics (OR=3.25, CI 1.78 to 5.94; p < 0.001). Strikingly though, those with problem behaviours identified at T1 were over 6 times more likely to have increased

For peer review only - http://bmjopen.bmj.com/site/about/guidelines.xhtml

prescribing of an antipsychotic (OR=6.45, CI 4.41 to 9.45; $p < 0.001$), over 3 times more likely for antidepressants (OR=3.44, CI 2.22 to 5.35; $p < 0.001$) and 3 times more likely for hypnotics/anxiolytics (OR=3.06, CI 1.72 to 5.44; $p < 0.001$).

The further regression investigating factors at T1 which are associated with prescribing at T2 (as opposed to change in prescribing reported in the paragraph above) shows that women were more likely to be prescribed antidepressants at T2, that older age had a small effect for antipsychotics and antidepressants at T2, a gradient across ability level for antiepileptics, and relationship with moderate and severe (but not profound) intellectual disabilities for antipsychotics at T2, and less antidepressants for people with profound intellectual disabilities. Mental ill-health and problem behaviours at T1 predicted prescribing of all classes.

For peer review only

Table 6: Multivariable analysis of T1 factors associated with changes in psychotropic prescriptions over time for the linked cohort (N=545)

	Antipsychotics		Antidepressants		Hypnotics/anxiolytics		Antiepileptics		Lithium*	
	OR (95% CI)	p-value	OR (95% CI)	p-value	OR (95% CI)	p-value	OR (95% CI)	p-value	OR (95% CI)	p-value
Time (continuous)	1.18 (0.87, 1.60)	0.280	2.80 (1.96, 4.00)	< 0.001	2.19 (1.34, 3.60)	0.002	1.40 (1.06, 1.84)	0.017	0.96 (0.26, 1.84)	0.4612
Male sex	0.99 (0.73, 1.34)	0.954	0.53 (0.37, 0.76)	< 0.001	0.83 (0.52, 1.35)	0.456	1.02 (0.77, 1.35)	0.896	0.93 (0.34, 2.59)	0.890
Age at T1	1.04 (1.03, 1.05)	< 0.001	1.02 (1.00, 1.03)	0.010	1.01 (0.99, 1.03)	0.260	0.99 (0.98, 1.00)	0.057	0.96(0.93, 0.99)	0.016
Level of intellectual disabilities	-	< 0.001	-	0.001	-	0.444	-	< 0.001	-	0.094
Mild	REF		REF		REF		REF		REF	
Moderate	1.88 (1.30, 2.74)	< 0.001	0.82 (0.54, 1.24)	0.346	1.64 (0.92, 2.92)	0.093	1.78 (1.26, 2.51)	< 0.001	0.23(0.07, 0.71)	0.011
Severe	2.49 (1.61, 3.85)	< 0.001	0.62 (0.37, 1.03)	0.063	1.12 (0.55, 2.31)	0.750	2.30 (1.55, 3.41)	< 0.001	1.49(0.17, 13.36)	0.721
Profound	1.61 (0.97, 2.69)	0.067	0.22 (0.11, 0.46)	< 0.001	1.24 (0.58, 2.62)	0.579	4.73 (3.07, 7.31)	< 0.001	0.46 (0.08, 2.64)	0.386

For peer review only - http://bmjopen.bmj.com/site/about/guidelines.xhtml

Mental ill-health**	4.11 (2.76, 6.11)	< 0.001	3.90 (2.53, 6.02)	< 0.001	3.25 (1.78, 5.94)	< 0.001	1.13 (0.76, 1.70)	0.547	-	-
Problem behaviours	6.45 (4.41, 9.45)	< 0.001	3.44 (2.22, 5.35)	< 0.001	3.06 (1.72, 5.44)	< 0.001	1.27 (0.90, 1.81)	0.174	-	-

*Mental illness and problem behaviours excluded from Lithium model due to small numbers **Not including problem behaviours

For peer review only

For peer review only - http://bmjopen.bmj.com/site/about/guidelines.xhtml

DISCUSSION

Principle findings

Despite numerous calls and guidelines for the withdrawal of antipsychotic drugs from people with intellectual disabilities who do not have psychosis/bipolar disorders, our linked cohort analysis demonstrates no progress over a decade. The comparison of the two cross-sectional whole cohorts does show a lower rate of antipsychotic prescribing in T2 than was observed in T1 but the rate is still high in T2, at 16.7% of the population. It appears that whilst people are not being withdrawn from antipsychotics once they commence them, new antipsychotic prescriptions are less commonly initiated than in the past. Over the decade, both the comparison of the whole cohort, and of the linked cohort, reveal a striking increase in the prescription of antidepressants (11.2% to 19.1%, and 9.9% to 22.2%). This was particularly so for women and for people with mild intellectual disabilities. To a lesser extent, there were also increases in prescribing of hypnotics/anxiolytics and antiepileptics in the linked cohort, but not in the comparison of the whole cohorts. This difference may be accounted for by the known increase in these prescriptions with age (5), which our study confirms, as the linked cohort is of course 10 years older in T2, whereas the ages and sex are similar in the whole cohorts in T2 and in T1. Whilst previous studies have reported high rates of antipsychotic prescribing, we are aware of none that have followed changes in prescribing rates over this length of time and related it to assessed mental ill-health.

Comparison with previous literature

To our knowledge only two studies have investigated longitudinal psychotropic prescribing patterns in community based samples of people with intellectual disabilities in the UK. Both studies were large and relied upon data extraction from primary care records. One reported antipsychotic prescribing for 17.1%, and antidepressants for 16.9%, with age being associated with both, and sex with antidepressants, similar to our T2 rates and findings. (4) The results of the other differed, reporting antipsychotic prescribing in 27.7% at the end of their period, but also found a fall of 4% per year over the study period, though no consistent trend for change in antidepressant prescriptions. (5) Neither study conducted psychiatric assessments on the population, limiting the precision of findings related to clinical diagnosis and GP recorded symptoms.

This study reaffirms the strong association between antipsychotic prescribing and problem behaviours reported in a number of other studies (21-24). However few studies have

For peer review only - http://bmjopen.bmj.com/site/about/guidelines.xhtml

separately reported associations between problem behaviours and antidepressants or hynotics/anxiolytics. An Irish study which investigated rates of prescribing of psychotropics in older adults with intellectual disabilities reported no increased risk of antidepressant prescribing or any association with problem behaviours (25).

Several studies have reported the increase in the rates of antidepressant prescribing in the general population across the UK, which our findings mirror. (26-28) In Scotland the number of antidepressant prescriptions rose from 1.16 to 3.53 million per year between 1992 and 2006, (29) and women receive more than men do. (26) The reasons for the increase are unclear and have been attributed to multiple factors such as the availability of newer classes of drugs with fewer side effects, improved management of depression, lack of availability of alternative interventions, (29) a widening of clinical uses (26) and patient expectations. Earlier studies have cited concerns that depression may have been underdiagnosed in the population with intellectual disabilities (30). One American study which retrospectively analysed outpatient psychiatric charts reported a higher than expected rate of antidepressant prescribing for the subgroup with intellectual disabilities and suggested this was indicative of increasing diagnosis of depressive disorders in adults with intellectual disabilities (31). Another US study analysed data from adults with intellectual disabilities living in community settings in New York State between 2006 and 2007 also reported a higher than expected rate of antidressant prescribing in this group (32). The substantial increase in antidepressant prescribing observed in the current study may indicate improved diagnosis in primary care for this population. (24) This study has also observed that problem behaviours were independently associated with antidepressant prescribing in adults with intellectual disabilities. However a systematic review of antidepressants and problem behaviours management in people with intellectual disabilities concluded that evidence of their effectiveness in this context is lacking (33). Longitudinal patterns of antidepressant prescribing require further investigation.

Implications for research and practice

This study has demonstrated that fewer new antipsychotic prescriptions are being initiated, but those prescribed antipsychotics in T1 were unlikely to have these drugs withdrawn over the next decade demonstrating possible reluctance from carers, families and individuals combined with a lack of evidence available to prescribers to direct cessation interventions (19, 34) . The issue therefore remains far from addressed, and the risks of long-term health

For peer review only - http://bmjopen.bmj.com/site/about/guidelines.xhtml

problems, death, and impact on quality of life associated with long-term antipsychotic prescriptions still needs further highlighting. (35) This study reinforces the need for frequent medication reviews for people with intellectual disabilities, alongside further research to investigate the long-term effects of antipsychotic medications on this population. (8) Further research to examine the barriers to antipsychotic drug reduction and to evaluate approaches to promoting reduction and withdrawal of antipsychotics for people with intellectual disabilities is needed. There is a dearth of evidence on antidepressant prescribing in the population with intellectual disabilities. The sharp increase in antidepressant prescribing observed in this study demands further research to understand the drivers for this. The association between increasing age and prescribing of antipsychotics and antidepressants also supports calls for research to investigate the implications of long-term psychotropic prescribing on older people with intellectual disabilities.(36)

What this paper adds

What is already known on this subject?

- There are high rates of antipsychotic prescribing in adults with intellectual disabilities
- Guidelines recommend reducing and withdrawing antipsychotic prescribing.
- Little longitudinal data is available to evidence whether progress is being made.

What this study adds?

- The proportion of adults with intellectual disabilities receiving antipsychotic prescriptions has fallen over the decade, though is still high at 16.7% of adults with intellectual disabilities.
- People are not being withdrawn from antipsychotics once commenced, whilst new antipsychotic prescriptions are less commonly initiated than in the past.
- There has been a striking increase in the prescription of antidepressants to 22%.

Competing interests

All authors have completed the ICMJE uniform disclosure form at www.icmje.org/coi_disclosure.pdf and declare: no support from any organisation for the submitted work; no financial relationships with any organisations that might have an interest

For peer review only - http://bmjopen.bmj.com/site/about/guidelines.xhtml

in the submitted work in the previous three years; no other relationships or activities that could appear to have influenced the submitted work .”

Author statement

AH and PMcS analysed the data, jointly interpreted it, AH wrote the first draft of the manuscript, AH, GovS-AC, DK, CM, and LA jointly conceived the project, interpreted the data and contributed to the manuscript. All authors approved the final version of the manuscript. S-AC is the study guarantor.

Funding

The study was funded by the Greater Glasgow Health Board, the Scottish Government via the Scottish Learning Disabilities Observatory and from the UK Medical Research Council (grant number: MC_PC_1717).

The study sponsors and funders had no role in the study design; in the collection, analysis, and interpretation of data; in the writing of the report; and in the decision to submit the article for publication.

The researchers are independent from the funders.

Statement

All authors had full access to all of the data (including statistical reports and tables) in the study and can take responsibility for the integrity of the data and the accuracy of the data analysis.

Transparency declaration

The manuscript's guarantor (S-AC) affirms that the manuscript is an honest, accurate, and transparent account of the study being reported; that no important aspects of the study have been omitted; and that there were no discrepancies from the study as planned.

Patient and Public Involvement

The Scottish Learning Disabilities Observatory has a steering committee which meets twice a year and provides strategic direction and oversight of all of the Observatory’s research,

For peer review only

including this project. The steering committee includes people with learning disabilities from "People First", a national group of self-advocates with learning disabilities.

Data sharing

No additional data is available

Acknowledgements

We are grateful to all the participants and their carers, to NHS Greater Glasgow learning disabilities primary care liaison team, to NHS Greater Glasgow and Clyde Safe Haven. This work uses data provided by patients and collected by the NHS as part of their care and support.

For peer review only

REFERENCES

1. Cooper SA, Smiley E, Morrison J, Williamson A, Allan L. Mental ill-health in adults with intellectual disabilities: prevalence and associated factors. Br J Psychiatry. 2007;190:27-35.
2. Cooper SA, Smiley E, Morrison J, Allan L, Williamson A, Finlayson J, et al. Psychosis and adults with intellectual disabilities - Prevalence, incidence, and related factors. Soc Psych Psych Epid. 2007;42(7):530-6.
3. Lunsky Y, Khuu W, Tadrous M, Vigod S, Cobigo V, Gomes T. Antipsychotic Use With and Without Comorbid Psychiatric Diagnosis Among Adults with Intellectual and Developmental Disabilities. Can J Psychiatry. 2017:706743717727240.
4. Lunsky Y, Khuu W, Tadrous M, Vigod S, Cobigo V, Gomes T. Antipsychotic Use With and Without Comorbid Psychiatric Diagnosis Among Adults with Intellectual and Developmental Disabilities. Can J Psychiat. 2018;63(6):361-9.
5. Glover G, Williams R. Prescribing of psychotropic drugs to people with learning disabilities and/or autism by general practitioners in England. London: Public Health England; 2015.
6. Kuijper Gd, Hoekstra P, Visser F, Scholte FA, Penning C, Evenhuis H. Use of antipsychotic drugs in individuals with intellectual disability (ID) in the Netherlands: prevalence and reasons for prescription. Journal of Intellectual Disability Research. 2010;54(7):659-67.
7. NICE. Challenging Behaviour and Learning Disabilities: Prevention and interventions for people with learning disabilities whose behaviour challenge. London: NICE; 2015.
8. Matson JL, Mahan S. Antipsychotic drug side effects for persons with intellectual disability. Res Dev Disabil. 2010;31(6):1570-6.
9. Doan TN, Lennox NG, Taylor-Gomez M, Ware RS. Medication use among Australian adults with intellectual disability in primary healthcare settings: A cross-sectional study. J Intellect Dev Dis. 2013;38(2):177-81.
10. Sheehan R, Hassiotis A, Walters K, Osborn D, Strydom A, Horsfall L. Mental illness, challenging behaviour, and psychotropic drug prescribing in people with intellectual disability: UK population based cohort study. BMJ. 2015;351:h4326.
11. Glassman AH, Bigger JT. Antipsychotic drugs: Prolonged QTc interval, torsade de pointes, and sudden death. Am J Psychiat. 2001;158(11):1774-82.
12. Cornett EM, Novitch M, Kaye AD, Kata V, Kaye AM. Medication-Induced Tardive Dyskinesia: A Review and Update. Ochsner J. 2017;17(2):162-72.
13. Stroup TS, Gray N. Management of common adverse effects of antipsychotic medications. World Psychiatry. 2018;17(3):341-56.
14. Tyrer P, Cooper SA, Hassiotis A. Drug treatments in people with intellectual disability and challenging behaviour. BMJ. 2014;349:g4323.
15. NICE. Mental health problems in people with learning disabilities: prevention, assessment and management. London: NICE; 2016.
16. Health Do. Transforming care: A national response to Winterbourne View Hospital:

Department of Health Review Final Report. 2012.
17. Disability FoPoI. Psychotropic drug prescribing for people with intellectual disability, mental health problems and/or behaviours that challenge: practice guidelines. London: The Royal College of Psychiatrists; 2016.
18. England N. Stopping over medication of people with a learning disability, autism or both (STOMP) 2019 [29/01/19]. Available from: https://www.england.nhs.uk/learning-disabilities/improving-health/stomp/.
19. Song M, Ware R, Doan TN, Harley D. Psychotropic medication use in adults with intellectual disability in Queensland, Australia, from 1999 to 2015: a cohort study. J Intellect Disabil Res. 2019.
20. Alvarez-Madrazo S, McTaggart S, Nangle C, Nicholson E, Bennie M. Data Resource Profile: The Scottish National Prescribing Information System (PIS). International Journal of Epidemiology. 2016;45(3):714-5f.
21. Sheehan R, Hassiotis A, Walters K, Osborn D, Strydom A, Horsfall L. Mental illness, challenging behaviour, and psychotropic drug prescribing in people with intellectual disability: UK population based cohort study. Bmj-Brit Med J. 2015;351.

For peer review only - http://bmjopen.bmj.com/site/about/guidelines.xhtml

22. O'Dwyer C, McCallion P, Henman M, et al. Prevalence and patterns of antipsychotic use and their associations with mental health and problem behaviours among older adults with intellectual disabilities. J Appl Res Intellect Disabil. 2019;32(4):981-93.
23. Deb S, Fraser W. The Use of Psychotropic Medication in People with Learning-Disability - Towards Rational Prescribing. Hum Psychopharm Clin. 1994;9(4):259-72.
24. Bowring DL, Totsika V, Hastings RP, Toogood S, McMahon M. Prevalence of psychotropic medication use and association with challenging behaviour in adults with an intellectual disability. A total population study. J Intellect Disabil Res. 2017;61(6):604-17.
25. Axmon A, El Mrayyan N, Eberhard J, Ahlstrom G. Pharmacotherapy for mood and anxiety disorders in older people with intellectual disability in comparison with the general population. BMC Psychiatry. 2019;19(1):238.
26. Lockhart P, Guthrie B. Trends in primary care antidepressant prescribing 1995-2007: a longitudinal population database analysis. Br J Gen Pract. 2011;61(590):e565-72.
27. Munoz-Arroyo R, Sutton M, Morrison J. Exploring potential explanations for the increase in antidepressant prescribing in Scotland using secondary analyses of routine data. Br J Gen Pract. 2006;56(527):423-8.
28. Hollinghurst S, Kessler D, Peters TJ, Gunnell D. Opportunity cost of antidepressant prescribing in England: analysis of routine data. Brit Med J. 2005;330(7498):999-1000.
29. Morrison J, Anderson MJ, Sutton M, Munoz-Arroyo R, McDonald S, Maxwell M, et al. Factors influencing variation in prescribing of antidepressants by general practices in Scotland. Br J Gen Pract. 2009;59(559):e25-31.
30. Robertson J, Emerson E, Gregory N, Hatton C, Kessissoglou S, Hallam A. Receipt of psychotropic medication by people with intellectual disability in residential settings. Journal of Intellectual Disability Research. 2000;44:666-76.
31. Hurley AD, Folstein M, Lam N. Patients with and without intellectual disability seeking outpatient psychiatric services: diagnoses and prescribing pattern. J Intellect Disabil Res. 2003;47(Pt 1):39-50.
32. Tsiouris JA, Kim SY, Brown WT, Pettinger J, Cohen IL. Prevalence of Psychotropic Drug Use in Adults with Intellectual Disability: Positive and Negative Findings from a Large Scale Study. Journal of Autism and Developmental Disorders. 2013;43(3):719-31.
33. Sohanpal SK, Deb S, Thomas C, Soni R, Lenotre L, Unwin G. The effectiveness of antidepressant medication in the management of behaviour problems in adults with intellectual disabilities: a systematic review. J Intellect Disabil Res. 2007;51(Pt 10):750-65.
34. McNamara R, Randell E, Gillespie D, Wood F, Felce D, Romeo R, et al. A pilot randomised controlled trial of community-led ANtipsychotic Drug REduction for Adults with Learning Disabilities. Health Technol Assess. 2017;21(47):1-92.
35. Scheifes A, Walraven S, Stolker JJ, Nijman HL, Egberts TC, Heerdink ER. Adverse events and the relation with quality of life in adults with intellectual disability and challenging behaviour using psychotropic drugs. Res Dev Disabil. 2016;49-50:13-21.
36. O'Dwyer M, Peklar J, Mulryan N, McCallion P, McCarron M, Henman MC. Prevalence, patterns and factors associated with psychotropic use in older adults with intellectual disabilities in Ireland. Journal of Intellectual Disability Research. 2017;61(10):969-83.

For peer review only - http://bmjopen.bmj.com/site/about/guidelines.xhtml

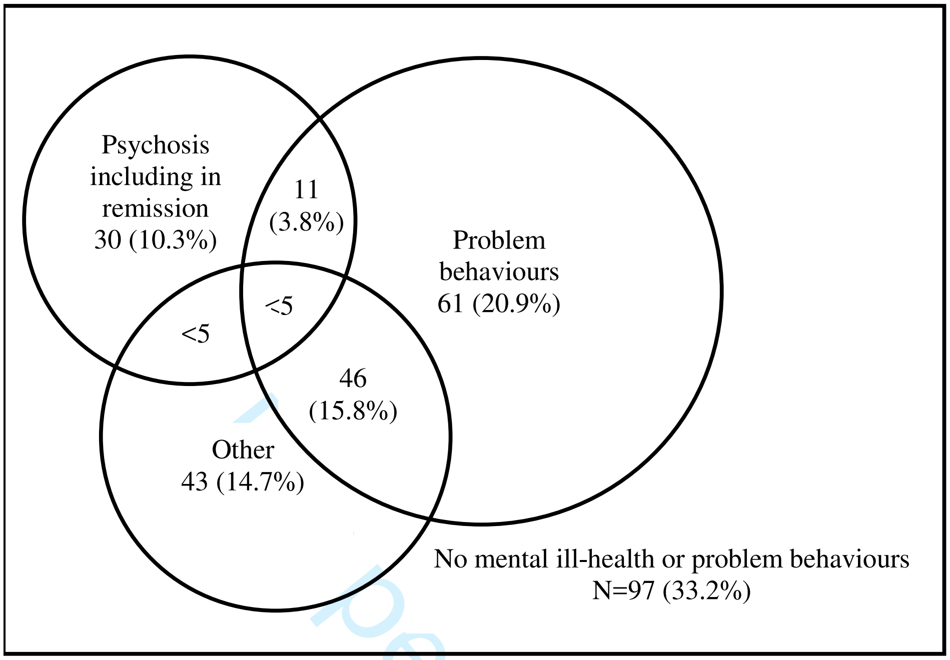

For peer review only

Supplementary figure 1: Participant flow diagram

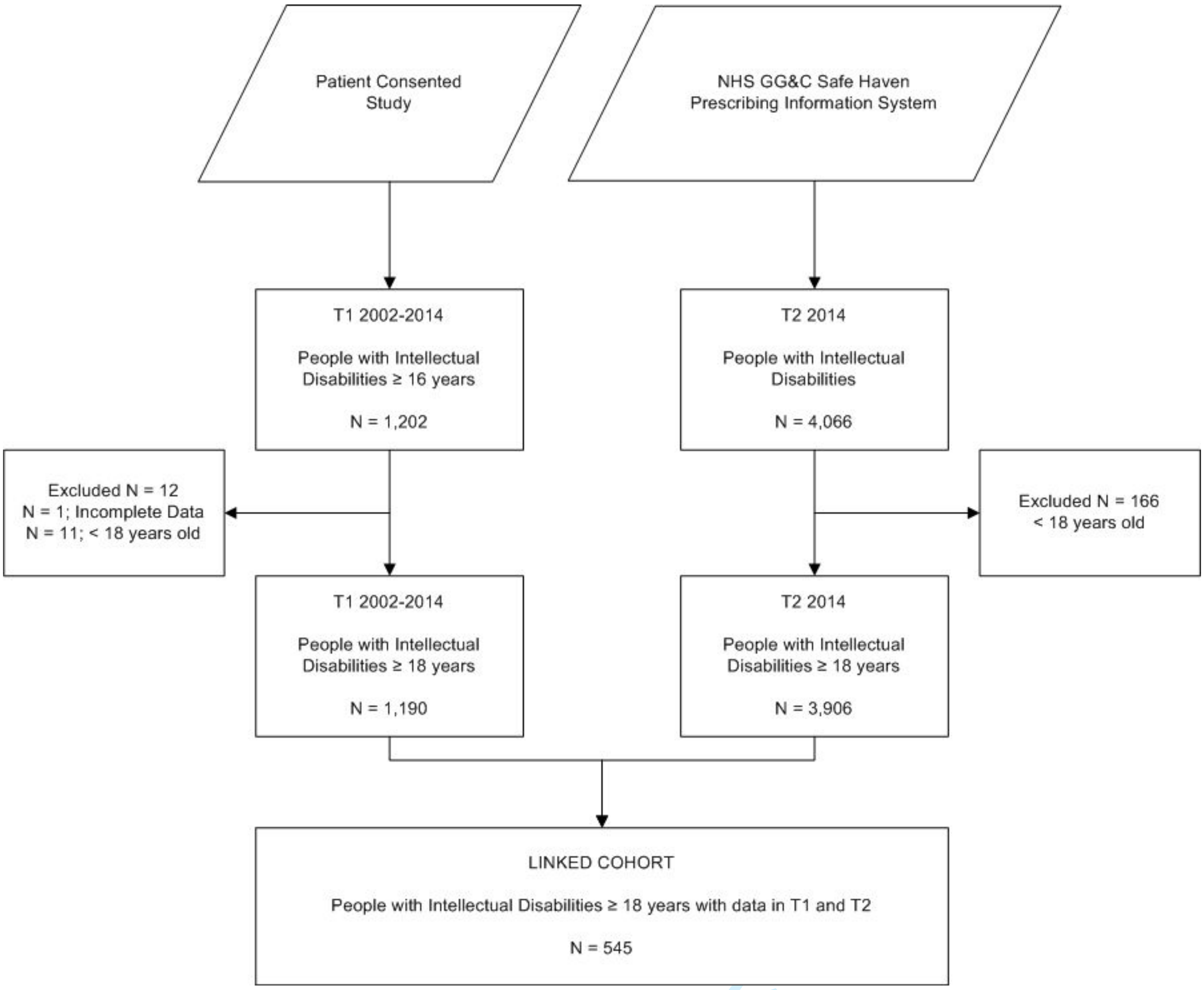

STROBE (Strengthening The Reporting of OBservational Studies in Epidemiology) Checklist

A checklist of items that should be included in reports of observational studies. You must report the page number in your manuscript where you consider each of the items listed in this checklist. If you have not included this information, either revise your manuscript accordingly before submitting or note N/A.

Note: An Explanation and Elaboration article discusses each checklist item and gives methodological background and published examples of transparent reporting. The STROBE checklist is best used in conjunction with this article (freely available on the Web sites of PLoS Medicine at http://www.plosmedicine.org/, Annals of Internal Medicine at http://www.annals.org/, and Epidemiology at http://www.epidem.com/). Information on the STROBE Initiative is available at www.strobe-statement.org.

Section and Item	Item No.	Recommendation	Reported on Page No.
**Title and Abstract**	1	(*a*) Indicate the study's design with a commonly used term in the title or the abstract	
		(*b*) Provide in the abstract an informative and balanced summary of what was done and what was found	
**Introduction**			
Background/Rationale	2	Explain the scientific background and rationale for the investigation being reported	
Objectives	3	State specific objectives, including any prespecified hypotheses	
**Methods**			
Study Design	4	Present key elements of study design early in the paper	
Setting	5	Describe the setting, locations, and relevant dates, including periods of recruitment, exposure, follow-up, and data collection	
Participants	6	(*a*) *Cohort study*—Give the eligibility criteria, and the sources and methods of selection of participants. Describe methods of follow-up<br>*Case-control study*—Give the eligibility criteria, and the sources and methods of case ascertainment and control selection. Give the rationale for the choice of cases and controls<br>*Cross-sectional study*—Give the eligibility criteria, and the sources and methods of selection of participants	
		(*b*) *Cohort study*—For matched studies, give matching criteria and number of exposed and unexposed<br>*Case-control study*—For matched studies, give matching criteria and the number of controls per case	
Variables	7	Clearly define all outcomes, exposures, predictors, potential confounders, and effect modifiers. Give diagnostic criteria, if applicable	

For peer review only

Section and Item	Item No.	Recommendation	Reported on Page No.
Data Sources/ Measurement	8*	For each variable of interest, give sources of data and details of methods of assessment (measurement). Describe comparability of assessment methods if there is more than one group	
Bias	9	Describe any efforts to address potential sources of bias	
Study Size	10	Explain how the study size was arrived at	
Quantitative Variables	11	Explain how quantitative variables were handled in the analyses. If applicable, describe which groupings were chosen and why	
Statistical Methods	12	(*a*) Describe all statistical methods, including those used to control for confounding	
		(*b*) Describe any methods used to examine subgroups and interactions	
		(*c*) Explain how missing data were addressed	
		(*d*) *Cohort study*—If applicable, explain how loss to follow-up was addressed<br>*Case-control study*—If applicable, explain how matching of cases and controls was addressed<br>*Cross-sectional study*—If applicable, describe analytical methods taking account of sampling strategy	
		(*e*) Describe any sensitivity analyses	
**Results**			
Participants	13*	(a) Report numbers of individuals at each stage of study—eg numbers potentially eligible, examined for eligibility, confirmed eligible, included in the study, completing follow-up, and analysed	
		(b) Give reasons for non-participation at each stage	
		(c) Consider use of a flow diagram	
Descriptive Data	14*	(a) Give characteristics of study participants (eg demographic, clinical, social) and information on exposures and potential confounders	
		(b) Indicate number of participants with missing data for each variable of interest	
		(c) *Cohort study*—Summarise follow-up time (eg, average and total amount)	
Outcome Data	15*	*Cohort study*—Report numbers of outcome events or summary measures over time	
		Case-control study—Report numbers in each exposure category, or summary measures of exposure	
		Cross-sectional study—Report numbers of outcome events or summary measures	

For peer review only

Section and Item	Item No.	Recommendation	Reported on Page No.
Main Results	16	(*a*) Give unadjusted estimates and, if applicable, confounder-adjusted estimates and their precision (eg, 95% confidence interval). Make clear which confounders were adjusted for and why they were included	
		(*b*) Report category boundaries when continuous variables were categorized	
		(*c*) If relevant, consider translating estimates of relative risk into absolute risk for a meaningful time period	
Other Analyses	17	Report other analyses done—eg analyses of subgroups and interactions, and sensitivity analyses	
**Discussion**			
Key Results	18	Summarise key results with reference to study objectives	
Limitations	19	Discuss limitations of the study, taking into account sources of potential bias or imprecision. Discuss both direction and magnitude of any potential bias	
Interpretation	20	Give a cautious overall interpretation of results considering objectives, limitations, multiplicity of analyses, results from similar studies, and other relevant evidence	
Generalisability	21	Discuss the generalisability (external validity) of the study results	
**Other Information**			
Funding	22	Give the source of funding and the role of the funders for the present study and, if applicable, for the original study on which the present article is based	

*Give information separately for cases and controls in case-control studies and, if applicable, for exposed and unexposed groups in cohort and cross-sectional studies.

Once you have completed this checklist, please save a copy and upload it as part of your submission. DO NOT include this checklist as part of the main manuscript document. It must be uploaded as a separate file.

For peer review only

BMJ Open

Changes over a decade in psychotropic prescribing for people with intellectual disabilities: prospective cohort study

Journal:	*BMJ Open*
Manuscript ID	bmjopen-2020-036862.R1
Article Type:	Original research
Date Submitted by the Author:	18-May-2020

Complete List of Authors:	Henderson, Angela; University of Glasgow, Institute of Health and Wellbeing<br>Mcskimming, Paula; University of Glasgow, Robertson Centre for Biostatistics<br>Kinnear, Deborah; University of Glasgow, Institute of Health and Wellbeing<br>McCowan, Colin ; University of Saint Andrews, School of Medicine<br>McIntosh, Alasdair; University of Glasgow, Robertson Centre for Biostatistics<br>Allan, Linda; University of Glasgow, Institute of Health and Wellbeing<br>Cooper, Sally-Ann; Glasgow University , Institute of Health and Wellbeing

**Primary Subject Heading**:	Mental health
Secondary Subject Heading:	Pharmacology and therapeutics
Keywords:	MENTAL HEALTH, PSYCHIATRY, CLINICAL PHARMACOLOGY, Adult psychiatry < PSYCHIATRY, Depression & mood disorders < PSYCHIATRY, Schizophrenia & psychotic disorders < PSYCHIATRY

SCHOLARONE™ Manuscripts

I, the Submitting Author has the right to grant and does grant on behalf of all authors of the Work (as defined in the below author licence), an exclusive licence and/or a non-exclusive licence for contributions from authors who are: i) UK Crown employees; ii) where BMJ has agreed a CC-BY licence shall apply, and/or iii) in accordance with the terms applicable for US Federal Government officers or employees acting as part of their official duties; on a worldwide, perpetual, irrevocable, royalty-free basis to BMJ Publishing Group Ltd ("BMJ") its licensees and where the relevant Journal is co-owned by BMJ to the co-owners of the Journal, to publish the Work in this journal and any other BMJ products and to exploit all rights, as set out in our licence.

The Submitting Author accepts and understands that any supply made under these terms is made by BMJ to the Submitting Author unless you are acting as an employee on behalf of your employer or a postgraduate student of an affiliated institution which is paying any applicable article publishing charge ("APC") for Open Access articles. Where the Submitting Author wishes to make the Work available on an Open Access basis (and intends to pay the relevant APC), the terms of reuse of such Open Access shall be governed by a Creative Commons licence – details of these licences and which Creative Commons licence will apply to this Work are set out in our licence referred to above.

Other than as permitted in any relevant BMJ Author's Self Archiving Policies, I confirm this Work has not been accepted for publication elsewhere, is not being considered for publication elsewhere and does not duplicate material already published. I confirm all authors consent to publication of this Work and authorise the granting of this licence.

For peer review only

Title: Changes over a decade in psychotropic prescribing for people with intellectual disabilities: prospective cohort study

Authors: Angela Henderson[1], Deputy Director, Scottish Learning Disabilities Observatory
Paula McSkimming[2], Biostatistician
Deborah Kinnear[1], Senior Lecturer
Colin McCowan[3], Professor in Health Data Science
Alasdair McIntosh[2], Junior Biostatistician
Linda Allan[1], Honorary Clinical Associate Professor
*Sally-Ann Cooper[1], Professor of Learning Disabilities

*Correspondence. Sally-Ann.Cooper@Glasgow.ac.uk 0141 211 0690

1. Institute of Health and Wellbeing, University of Glasgow, Mental Health and Wellbeing research group, 1st Floor, Administrative Building, Gartnavel Royal Hospital, 1055 Great Western Road, Glasgow, G12 0XH
2. Robertson Centre for Biostatistics, Institute of Health and Wellbeing, University of Glasgow, Boyd Orr Building, University Avenue, Glasgow, G12 8QQ
3. School of Medicine, University of St Andrews, North Haugh, St Andrews KY16 9TF

Word count: 3,927

For peer review only

For peer review only - http://bmjopen.bmj.com/site/about/guidelines.xhtml

ABSTRACT

OBJECTIVES: To investigate psychotropic prescribing in the intellectual disabilities population over 10 years, and associated mental ill-health diagnoses.

DESIGN: (a) Comparison of cross-sectional data in 2002-2004 (T1), and 2014 (T2). (b) Longitudinal cohort study with detailed health assessments at T1, and record linkage to T2 prescribing data.

SETTING: General community.

PARTICIPANTS: (a) 1,190 adults with intellectual disabilities in T1 compared with 3,906 adults with intellectual disabilities in T2. (b) 545/1,190 adults with intellectual disabilities in T1 were alive and their records linked to T2 prescribing data.

MAIN OUTCOME MEASURES: Encashed regular and as-required psychotropic prescriptions.

RESULTS: (a) 50.7% (603/1,190) in T1, and 48.2% (1,881/3,906) in T2 were prescribed 1+ psychotropics: antipsychotics 24.5% (292/1,190) in T1 and 16.7% (653/3,906) in T2, antidepressants 11.2% (133/1,190) in T1 and 19.1% (746/3,906) in T2. 30.0% (62/292) prescribed antipsychotics in T1 had psychosis or bipolar disorder, 33.2% (97/292) had no mental ill-health or problem behaviours, 20.6% (60/292) had problem behaviours but no psychosis/bipolar disorder. (b) Psychotropics increased from 47.0% (256/545) in T1 to 57.8% (315/545) in T2 ($p<0.001$): antipsychotics did not change (OR=1.18; CI (0.87, 1.60); p=0.280), there was an increase for antidepressants (OR=2.80; CI 1.96, 4.00; $p<0.001$), hypnotics/anxiolytics (OR=2.19; CI 1.34, 3.61; p=0.002), and antiepileptics (OR=1.40; CI 1.06, 1.84; p=0.017). Antipsychotic prescribing increased for people with problem behaviours in T1 (OR=6.45, CI 4.41, 9.45; $p<0.001$), more so than for people with other mental ill-health in T1 (OR 4.11, CI 2.76, 6.11; $p<0.001$).

CONCLUSIONS: Despite concerns about antipsychotic prescribing and guidelines recommending their withdrawal, it appears that whilst fewer antipsychotic prescriptions were initiated by T2 than in T1, people were not withdrawn from them once commenced. People with problem behaviours had increased prescribing. There was also a striking increase in antidepressant prescriptions. Adults with intellectual disabilities need frequent and careful medication reviews.

Key words: intellectual disabilities, psychotropics, antipsychotics, antidepressants, hypnotics, anxiolytics, anti-epileptics, lithium, mental ill-health

For peer review only - http://bmjopen.bmj.com/site/about/guidelines.xhtml

Article summary

Strengths and limitations

The strengths of this study are:

- The large cohort size, longitudinal design, detailed ascertainment of the population with intellectual disabilities, and the detailed health assessments at T1.
- The cross-sectional cohorts were population-based at T1 and T2, and representative of the population with intellectual disabilities; the linked cohort was similar in characteristics to the cross-sectional cohort at T1, suggesting it is also representative and therefore that the results are generalisable.

The limitations of this study are:

- Only 73% of general practices agreed to data extraction, and this combined with deaths are likely to be the main reasons for 545/1,190 of the participants being linked in the T2 data, 10 years later.
- The different methods of data collection, with specialist individual assessments at T1 and electronic data extraction at T2; in particular there is a large proportion of missing information and may be inaccuracies on recorded level of intellectual disabilities in the general practitioner data at T2 limiting comparability of this variable between the T1 and T2 cohorts.
- The study did not investigate changes in dosages, polypharmacy or duration of use and there is a lack of mental ill-health data at T2.

INTRODUCTION

Mental ill-health is common in people with intellectual disabilities. (1) The prevalence of psychosis in this population is reported to be around 4% based on cross-sectional data, and the rate of people with a first psychotic episode is about 10 times that of the general population. (2) Whilst the rates of psychosis are relatively high, antipsychotics are often prescribed for adults with intellectual disabilities who do not have a record of severe mental ill-health (3, 4), often for problem behaviours, (5-9) and despite limited evidence to support their use beyond short-term sedation. (7) Indeed, 71% of people with intellectual disabilities who are prescribed antipsychotics have been reported to have no record of serious mental ill-health. (10) This is important, as antipsychotics have numerous disabling, painful, and disfiguring side effects, some of which are life threatening such as tardive dyskinesia, cardiac arrhythmias, and sudden cardiac death. (11-13) Antipsychotics are also frequently prescribed

For peer review only - http://bmjopen.bmj.com/site/about/guidelines.xhtml

for children and young people with a range of developmental disabilities and problem behaviours (14, 15), and in the young general population, rates increase during adolescence. (16)

Concerns have repeatedly been raised about the overuse of antipsychotics, and the need for more proportionate prescribing for people with intellectual disabilities. (7, 17-19) In 2016 a national campaign was launched by NHS England in partnership with the Royal Colleges of General Practitioners, Psychiatrists, and Nursing, the Royal Pharmaceutical Society, and British Psychological Society to address these concerns in England: "Stopping over medication of people with a learning disability, autism or both (STOMP)". Guidelines from STOMP, the National Institute for Health and Care Excellence, and the Royal College of Psychiatrists highlight that prescribers, where appropriate, should reduce or withdraw antipsychotics for people with intellectual disabilities who do not have psychosis. (7, 20, 21) However, there is very little empirical evidence from the UK on any changes in antipsychotic prescribing patterns over time. An exception is a study by Sheehan et al (2015) that extracted data from general practice records on 33,016 adults with a record of intellectual disabilities, with a median follow-up of 5.5 years. (10) They reported the incidence of new psychotropic prescription to be 518/10,000 person years. Prescriptions of antipsychotics fell by 4% per year over the study period, as did mood stabilisers, whilst there was no consistent trend for antidepressants or anxiolytics/hypnotics. Sheehan et al. (2015) reported that 47% of those with "challenging behaviour" had received antipsychotic drugs, but only 12% had a record of severe mental ill-health, and that 26% of those prescribed antipsychotics did not have a record of severe mental ill-health or "challenging behaviour". A limitation of this study is in the identification of 'challenging behaviour' through a heterogeneous list of 45 Read codes (the system used in general practices in the UK to code diagnoses). Read codes do not provide a robust method for ascertaining problem behaviours. Additionally incomplete and variable recording practices do not always accurately reflect a person's health. (10) Another study from Australia investigated psychotropic medication use between 1999 and 2015 in a cohort of 138 participants (22) and also found a strong association between problem behaviours and psychotropic medication. In this cohort the study reported that once psychotropic medications were prescribed they were unlikely to be removed, and observed little change in prescribing of antipsychotics between 1999 and 2015 (24/138 (24%) to 23/92 (23%)). A sharp increase in the prescribing of antidepressants from 16.7% to 36.1% across the same period was also observed. However, whilst this was a longitudinal cohort not all

For peer review only - http://bmjopen.bmj.com/site/about/guidelines.xhtml

participants took part in all waves of data collection, therefore it is not possible to ascertain within group changes in prescribing.

Aim

The aim of this study is to investigate changes over a decade in psychotropic prescribing for adults with intellectual disabilities, and the diagnoses associated with antipsychotics, from detailed psychiatric assessments.

METHODS

Ethical approval

NHS Greater Glasgow Primary Care Trust, Community & Mental Health Research Ethics Committee granted ethical approval (project number 01/44). Between 2002-2004 (T1), individual consent to participate was taken in line with Scottish law. In 2014 (T2), 191/263 (73%) general practices in NHS Greater Glasgow and Clyde area participated, and the NHS Greater Glasgow and Clyde Local Privacy Advisory Committee approved electronic extraction and linkage of primary care records.

Participants

In 2000-2001, a primary care intellectual disabilities register was established of adults with intellectual disabilities, aged ≥16 years, living in the NHS Greater Glasgow area. This was delivered through partnership between the intellectual disabilities clinical service and all general practitioners in the area. People with intellectual disabilities were identified through social work services for people with intellectual disabilities; local authority funding arrangements for people receiving paid support of any kind, including day opportunities; local specialist health services for people with intellectual disabilities; the Health Board; and general practices who were financially incentivised to identify their registered patients with intellectual disabilities (100% of general practices participated). Intellectual disabilities nurses reviewed all cases on the register to ascertain if intellectual disabilities were present, those that did not have intellectual disabilities were removed from the register. The register was then updated annually by the general practices and the intellectual disabilities clinical service. Between 2002-2004, the register was used to invite people living in a *representative part of the Health Board area* to partipate in the study, 67% agreed to do so: these participants were recruited to a longitudinal cohort between 2002 and 2004 (T1), and had detailed health assessments at that time. 1,190 were aged ≥18 years, and comprise the study

For peer review only

population reported here. In 2014 (T2), for people on the register and living in *the whole of the Health Board area,* data was extracted from primary care records; 73% of general practices in the Health Board agreed to the data extraction, data was extracted on their 3,906 patients with intellectual disabilities aged ≥18 years, who comprise the study population reported here.

Process and measures

Semi-structured individual health assessments, including medication review, assessment of level of intellectual disabilities (via structured questions on abilities, and the Vineland Scale (23)), mental ill-health symptoms including problem behaviours and autism, were conducted at T1 by one of six intellectual disabilities nurses and one of three general practitioners with a special interest in intellectual disabilities. These were preceeded by review and data collection from the person's general practitioner medical records, and then conducted with the person with intellectual disabilities and their carer(s). This included a review of drug charts for the participants in supported care. The 54% of individuals identified with possible, probable, or definite mental ill-health (including problem behaviours and autism) were then assessed by the study's psychiatrists who were specialists in intellectual disabilities psychiatry. Information from each person's psychiatric assessment was reviewed by two psychiatrists who case-conferenced and agreed the classification of the mental ill-health, using ICD-10-DCR (24), DSM-IV-TR (25), DC-LD (26) and clinical criteria. Details have been previously reported. (1) Given that ICD-10 and DSM criteria function poorly for adults with moderate to severe intellectual disabilities (particularly with regards to problem behaviours), in this study we report the clinical diagnoses agreed together by the study psychiatrists. Data collection was over a two year period. Drugs were coded using British National Formulary (BNF) codes.

At T2, the 3,906 adults with intellectual disabilities identified from primary care records were record linked to Prescribing Information System (PIS) data, using the Community Health Index (CHI), which is the NHS patient identification number, unique to each person. PIS is Scotland's electronic record of all encashed prescriptions (i.e. not prescriptions issued, or drugs administered, but those that the carers/person with intellectual disabilities actually took to a pharmacist and exchanged for the drugs), and includes a record of the BNF code of each prescribed drug. (27) Prescribing information was then extracted using BNF codes for the 3,906 adults with intellectual disabilities to identify all prescriptions of antipsychotics,

For peer review only - http://bmjopen.bmj.com/site/about/guidelines.xhtml

antidepressants, antiepileptics, lithium, and hypnotics/anxiolytics across a specific 12-week prescribing window in 2014, including both regular prescriptions and as-required medication. To establish the longitudinal cohort the CHI number was used to identify T1 participants in the T2 dataset, enabling comparison of encashed medications across the decade. Only participants with complete data who were aged 18 years and over were included in the analyses (Figure 1).

Statistical analysis

Subject characteristics and prescribing information were summarised descriptively with mean and standard deviation (SD) for continuous outcomes and number and percentage for categorical outcomes at each time point (T1 and T2). Prescribing information at each time point was summarised using binary variables for each class of medications of interest (yes/no), allowing prescribing patterns to be investigated between the two time points in the study using McNemar's tests on the subset of the linked cohort, for whom there were prescribing records at both T1 and T2. This analysis was extended to explore whether there were associations between time or the subject characteristics at T1, with each prescription category using binary logistic regression models. Each model included multiple explanatory variables, specifically; time as a binary variable to indicate each time point T1 and T2; sex; age as a continuous measure; level of intellectual disabilities as four-level categorical variable; presence of mental ill-health (yes/no, excluding problem behaviours); having problem behaviours (yes/no); and a binary dependant variable for each class of medication (yes/no). Logistic regression models were also fitted with the above T1 subject characteristics to explore their association with each prescribing category specifically at T2. Odd ratios are reported for all logistic regression models with corresponding 95% confidence intervals (CIs) and p-values. A p-value of less than 0.05 is considered as statistically significant. Statistical analyses were conducted using SAS version 9.3.

Patient and Public Involvement

The Scottish Learning Disabilities Observatory has a steering committee which meets twice a year and provides strategic direction and oversight of all of the Observatory's research, including this project. The steering committee includes people with learning disabilities from 'People First', a national group of self-advocates with intellectual disabilities.

RESULTS

For peer review only - http://bmjopen.bmj.com/site/about/guidelines.xhtml

Participant characteristics of the cross-sectional cohorts

Data for those who had incomplete data at T1 and for those who were aged under 18 years at either time point were excluded from further analyses. Table 1 shows participant characteristics; age, sex, level of intellectual disabilities at T1 (n=1,190) and T2 (n=3,906), and mental ill-health and epilepsy diagnoses at T1. No mental health or epilepsy data were available at T2.

Table 1. Participant characteristics for the cross-sectional cohorts at T1 and T2

Characteristic	T1 aged 18+years (N=1,190)	T2 aged 18+ years (N=3,906)
**Age Mean (SD)**	44.6 (14.3)	45.4 (15.5)
**Sex N(%):**		
Male	671 (56.4)	2,260 (57.9)
Female	519 (43.6)	1,646 (42.1)
**Level of intellectual disabilities N(%):**		
Mild	451 (37.9)	1047 (26.8)
Moderate	319 (26.8)	859 (22.0)
Severe	233 (19.6)	595 (15.2)
Profound	187 (15.7)	197 (5.0)
Unknown	0	1,208 (30.9)
**Epilepsy N(%)**	419 (35.2%)	Not collected
**Type of mental ill-health N(%):**		
Psychosis, including psychosis in remission	52 (4.4)	Not collected
Problem behaviours	244 (20.5)	
Autism	80 (6.7)	
ADHD	15 (1.3)	
Unipolar depression	51 (4.3)	
Bipolar disorder	21 (1.8)	
Anxiety disorders	32 (2.7)	
Organic disorder	20 (1.7)	
Personality disorder	9 (0.8)	
Obsessive compulsive disorder	7 (0.6)	
Psychosexual disorder	< 5	
Other	15 (1.3)	
Mental ill-health (including problem behaviours)	438 (36.8)	
Mental ill-health (excluding problem behaviours)	194 (16.3)	

Prescribing for the cross-sectional cohorts

At least one psychotropic was prescribed at T1 for 50.7% (603/1,190), and at T2 for 48.2% (1,881/3,906) (table 2) of participants. At T1, 24.5% (292/1,190) of participants were prescribed antipsychotics and at T2 16.7% (653/3,906) were prescribed antidepressants. At T1, antidepressants were prescribed for 11.2% (133/1,190), and at T2 for 19.1% (746/3,906)

For peer review only - http://bmjopen.bmj.com/site/about/guidelines.xhtml

of participants. Hypnotic/anxiolytic, lithium, and anti-epileptic prescribing was similar at T1 and T2.

The types of mental ill-health experienced by the 292 participants at T1 who were taking antipsychotics are shown in table 3. The most common diagnosis within this group was problem behaviours at 40.8% (119/292). Of note, 33.2% (97/292) of the people taking antipsychotics did not have any identified mental ill-health or problem behaviours. Figure 2 demonstrates the overlap between groups of the people who were taking antipsychotics at T1 and selected diagnoses.

Figure 2. Types of mental ill-health experienced by people prescribed antipsychotics at T1 n=292

Table 3 also shows the types of mental ill-health experienced by the 230 participants at T1 who were taking antipsychotics, after excluding people with psychosis (or psychosis in remission) or bipolar disorder (given that they would be expected to be prescribed antipsychotics, and given the considerable overlap between disorders shown in Figure 2). Most strikingly, 97/230 (42.2%) on antipsychotics had no mental ill health or problem behaviours. The proportion of people in each diagnostic category and without co-occurring psychosis or bipolar disorder who were taking antipsychotics was considerable for all types: 11.7% (27/230) for autism, 7.0% (16/230) for unipolar depression, 2.6% (6/229) for anxiety disorders and 2.2% (5/230) or less for all other diagnoses.

Table 2. Psychotropics prescribed for the cross-sectional cohorts at T1 and T2

Prescriptions	T1 aged 18+ years (N=1,190)	T2 aged 18+ years (N=3, 906)
Any psychotropic drug	603 (50.7)	1,881 (48.2)
Antipsychotics	292 (24.5)	653 (16.7)
Antidepressants	133 (11.2)	746 (19.1)
Antiepileptics	333 (28.0)	1,028 (26.3)
Lithium	14 (1.2)	31 (0.8)
Hypnotics/anxiolytics	81 (6.8)	305 (7.8)
Missing data	0	3 (0.1)

Table 3. Types of mental ill-health at T1 experienced by people prescribed antipsychotics at T1, and after excluding people with psychosis and bipolar disorder

For peer review only - http://bmjopen.bmj.com/site/about/guidelines.xhtml

Mental ill-health at T1		Adults (18+ years) taking antipsychotics at T1	
**Diagnosis**	**All (N=1,190)**	**All (N=292)**	**Excluding people with psychosis and bipolar disorder (N=230)**
Psychosis, including psychosis in remission	52	45 (15.4)	-
Problem behaviours	244	119 (40.8)	100 (43.5)
Autism	80	30 (10.3)	27 (11.7)
ADHD	15	12 (4.1)	11 (4.8)
Unipolar depression	51	20 (6.9)	16 (7.0)
Bipolar disorder	21	17(5.8)	-
Anxiety disorders	32	7 (2.4)	6 (2.6)
Organic disorder	20	5 (1.7)	< 5
Personality disorder	9	5 (1.7)	< 5
Obsessive Compulsive Disorder	7	3 (1.0)	< 5
Psychosexual disorder	< 5	< 5	< 5
Other	15	6 (2.1)	5 (2.2)
Mental ill-health (including problem behaviours)	438	195 (66.8)	133 (57.8)
Mental ill-health (excluding problem behaviours)	194	76 (26.3)	33 (14.4)
No mental ill-health nor problem behaviours	752	97 (33.2)	97 (42.2)

- **Insert figure 2 here. Types of mental ill-health experienced by people prescribed antipsychotics at T1 -**

Participant characteristics of the longitudinal, linked cohort

The longitudinal, linked cohort included the 545 adults who were in the T1 cohort and who were also identified within the GP records at T2. Table 4 shows their age, sex, level of intellectual disabilities, epilepsy diagnosis, and mental ill-health at T1. They appear to be broadly representative of the whole cohort at T1 on these characteristics.

Table 4. Participant characteristics at T1 for people in the longitudinal cohort

Characteristic	T1 (aged 18+ years) N=545 (%)
**Age Mean (SD)**	41.8 (13.2)

For peer review only - http://bmjopen.bmj.com/site/about/guidelines.xhtml

**Sex N (%):**	
Male	322 (59.1)
Female	223 (40.9)
**Level of intellectual disabilities N (%):**	
Mild	237 (43.5)
Moderate	154 (28.3)
Severe	89 (16.3)
Profound	65 (11.9)
**Epilepsy**	173 (31.7)
**Type of mental ill-health N (%):**	
Psychosis, including psychosis in remission	32 (5.9)
Problem behaviours	109 (20.0)
Autism	38 (7.0)
ADHD	8 (1.5)
Unipolar depression	23 (4.2)
Bipolar disorder	10 (1.8)
Anxiety disorders	14 (2.6)
Organic disorder	< 5
Personality disorder	7 (1.3)
Obsessive compulsive disorder	< 5
Psychosexual disorder	<5
Other	6 (1.1)
Mental ill-health (including problem behaviours)	190 (34.9)
Mental ill-health (excluding problem behaviours)	81 (14.9)

Prescribing for the longitudinal, linked cohort

At least one psychotropic medication was prescribed at T1 for 47.0% (256/545), and for 57.8% (315/545) at T2 (table 5) which is a significant increase over time (p<0.001). Antidepressants were prescribed for 9.9% (54/545) of participants at T1, and at T2 for 22.0% (120/545), showing a significant increase (p<0.001). At T1, hypnotics/anxiolytics were prescribed for 4.6% (25/545) of participants, and at T2 for 9.4% (51/545), a significant increase (p<0.001). At T1, antiepileptics were prescribed for 24.8% (135/545) of participants, and at T2 for 31.0% (169/545), a significant increase (p<0.001). Prescribing patterns levels at T1 and T2 were similar for both antipsychotics and lithium. Of the 128 people prescribed antipsychotics at T1, 77.3% (99/128) were prescribed antipsychotics at T2; only 29 (22.7%) had been withdrawn, and 43/545 (7.9%) had started on an antipsychotic between the two timepoints.

Table 5. Psychotropic medications prescribed for the longitudinal, linked cohort at T1 and T2

Medication group	T1	T2	P-value

For peer review only - http://bmjopen.bmj.com/site/about/guidelines.xhtml

	(N=545)	(545)	
**Any psychotropic medication**	256 (47.0%)	315 (57.8%)	p<0.001
**Antipsychotics**	128 (23.5%)	142 (26.1%)	p=0.099
**Antidepressants**	54 (9.9%)	120 (22.0%)	p<0.001
**Hypnotics/anxiolytics**	25 (4.6%)	51 (9.4%)	P<0.001
**Antiepileptics**	135 (24.8%)	169 (31.0%)	P<0.001
**Lithium**	7 (1.3%)	10 (1.8%)	P=0.180

The logistic regression analyses, taking account of sex, age, level of intellectual disabilities, having mental ill-health (excluding problem behaviours) and having problem behaviours at T1 (Table 6) shows no significant difference in antipsychotic prescribing rate in the linked cohort over the decade (OR = 1.18; CI 0.87 to 1.60; p = 0.280), an increase in antidepressants (OR=2.80; CI 1.96 to 4.00; p<0.001), hypnotics/anxiolytics (OR=2.19; CI 1.34 to 3.6; p=0.002), and antiepileptic prescribing (OR=1.40; CI 1.06 to 1.84; p = 0.017). Sex was not independently associated with prescribing, except that women were more likely to have an increase in antidepressants than men after adjusting for time (OR = 0.53, CI 0.37 to 0.78; p<0.001). Older age had a small effect on prescribing for antipsychotics and antidepressants. Effects are also observed for level of intellectual disabilities. There was a gradient for antiepileptics (increased prescribing with increasing severity of intellectual disabilities) and a gradient for antidepressants (reduced prescribing with increasing severity of intellectual disabilities). However there was no gradient across different ability levels for antipsychotic prescribing. As expected, participants with a diagnosed mental health problem (excluding problem behaviours) at T1 were more likely to be prescribed antipsychotics (OR=4.11, CI 2.76 to 6.11; p < 0.001), antidepressants (OR=3.90, CI 2.53 to 6.02; p < 0.001), and hypnotics/anxiolytics (OR=3.25, CI 1.78 to 5.94; p < 0.001). Strikingly though, those with problem behaviours identified at T1 were over 6 times more likely to have increased prescribing of an antipsychotic (OR=6.45, CI 4.41 to 9.45; p < 0.001), over 3 times more likely for antidepressants (OR=3.44, CI 2.22 to 5.35; p < 0.001) and 3 times more likely for hypnotics/anxiolytics (OR=3.06, CI 1.72 to 5.44; p < 0.001).

The further regression (Supplementary table 1) investigating factors at T1 which are associated with prescribing at T2 (as opposed to change in prescribing reported in the

For peer review only - http://bmjopen.bmj.com/site/about/guidelines.xhtml

paragraph above) shows that women were more likely to be prescribed antidepressants at T2, that older age had a small effect for antipsychotics and antidepressants at T2, a gradient across ability level for antiepileptics, and relationship with moderate and severe (but not profound) intellectual disabilities for antipsychotics at T2, and less antidepressants for people with profound intellectual disabilities. Mental ill-health and problem behaviours at T1 predicted prescribing of all classes.

For peer review only

For peer review only - http://bmjopen.bmj.com/site/about/guidelines.xhtml

Table 6: Multivariable analysis of exploratory T1 factors and time with psychotropic prescriptions for the linked cohort (N=545)

	Antipsychotics		Antidepressants		Hypnotics/anxiolytics		Antiepileptics		Lithium*	
	OR (95% CI)	p-value	OR (95% CI)	p-value	OR (95% CI)	p-value	OR (95% CI)	p-value	OR (95% CI)	p-value
Time	1.18 (0.87, 1.60)	0.280	2.80 (1.96, 4.00)	< 0.001	2.19 (1.34, 3.60)	0.002	1.40 (1.06, 1.84)	0.017	0.69 (0.26, 1.84)	0.4612
Male sex	0.99 (0.73, 1.34)	0.954	0.53 (0.37, 0.76)	< 0.001	0.83 (0.52, 1.35)	0.456	1.02 (0.77, 1.35)	0.896	0.93 (0.34, 2.59)	0.890
Age at T1	1.04 (1.03, 1.05)	< 0.001	1.02 (1.00, 1.03)	0.010	1.01 (0.99, 1.03)	0.260	0.99 (0.98, 1.00)	0.057	0.96(0.93, 0.99)	0.016
Level of intellectual disabilities (compared with Mild ID)	-	< 0.001	-	0.001	-	0.444	-	< 0.001	-	0.094
Moderate	1.88 (1.30, 2.74)	< 0.001	0.82 (0.54, 1.24)	0.346	1.64 (0.92, 2.92)	0.093	1.78 (1.26, 2.51)	< 0.001	0.23(0.07, 0.71)	0.011
Severe	2.49 (1.61, 3.85)	< 0.001	0.62 (0.37, 1.03)	0.063	1.12 (0.55, 2.31)	0.750	2.30 (1.55, 3.41)	< 0.001	1.49(0.17, 13.36)	0.721
Profound	1.61 (0.97, 2.69)	0.067	0.22 (0.11, 0.46)	< 0.001	1.24 (0.58, 2.62)	0.579	4.73 (3.07, 7.31)	< 0.001	0.46 (0.08, 2.64)	0.386
Mental ill-health**	4.11 (2.76,	< 0.001	3.90 (2.53,	< 0.001	3.25 (1.78,	< 0.001	1.13 (0.76,	0.547	-	-

For peer review only - http://bmjopen.bmj.com/site/about/guidelines.xhtml

	6.11)		6.02)		5.94)		1.70)			
Problem behaviours	6.45 (4.41, 9.45)	< 0.001	3.44 (2.22, 5.35)	< 0.001	3.06 (1.72, 5.44)	< 0.001	1.27 (0.90, 1.81)	0.174	-	-

*Mental illness and problem behaviours excluded from Lithium model due to small numbers **Not including problem behaviours

For peer review only

DISCUSSION

Principal findings

Despite numerous calls and guidelines in the UK for the withdrawal of antipsychotic drugs from people with intellectual disabilities who do not have psychosis/bipolar disorders, (7, 20, 21) our longitudinal, linked cohort analysis demonstrates no progress over a decade. The comparison of the two cross-sectional cohorts does show a lower rate of antipsychotic prescribing in T2 than was observed in T1 but the rate is still high in T2, at 16.7% of the population. It appears that whilst few people are being withdrawn from antipsychotics once they commence them, new antipsychotic prescriptions are less commonly initiated than in the past. Over the decade, comparison of both the cross-sectional cohorts, and of the longitudinal, linked cohort, reveal a striking increase in the prescription of antidepressants (11.2% to 19.1%, and 9.9% to 22.0%). This was particularly so for women and for people with mild intellectual disabilities. To a lesser extent, there were also increases in prescribing of hypnotics/anxiolytics and antiepileptics in the linked cohort, but not in the comparison of the cross-sectional cohorts. This difference may be accounted for by the known increase in these prescriptions with age (5), as the linked cohort is of course 10 years older in T2, whereas the ages and sex are similar in the whole cohorts in T2 and in T1. The age-related change in antiepileptic prescribing in the longitudinal linked cohort, but not in the comparison of the similarly aged cross-sectional cohorts, contextualises the antipsychotic and antidepressant findings (prescribing trends in general), as antiepileptics were almost all prescribed for the highly prevalent condition of epilepsy in this population. Whilst previous studies have reported high rates of antipsychotic prescribing, we are aware of none that have investigated prescribing over this length of time and related fluctuations to assessed mental ill-health.

Comparison with previous literature

To our knowledge only two studies have investigated longitudinal psychotropic prescribing patterns in community based samples of people with intellectual disabilities in the UK. Both studies were large and relied upon data extracted from primary care records. One reported antipsychotic prescribing for 17.1%, and antidepressants for 16.9% of adults with intellectual disabilities, with age being associated with both, and sex with antidepressants, similar to our T2 results. (5) The results of the other differed, reporting antipsychotic prescribing in 27.7% of participants at the end of their study period, but also found a fall of 4% per year over the whole study period, though no consistent trend antidepressant prescriptions was reported. (5)

For peer review only - http://bmjopen.bmj.com/site/about/guidelines.xhtml

Neither study conducted psychiatric assessments on the population, limiting the precision of findings related to clinical diagnosis and GP recorded symptoms.

This study reaffirms the strong association between antipsychotic prescribing and problem behaviours reported in a number of other studies (28-31). However few studies have separately reported associations between problem behaviours and antidepressants or hynotics/anxiolytics. An Irish study which investigated rates of prescribing of psychotropics in older adults with intellectual disabilities reported no increased risk of antidepressant prescribing or any association with problem behaviours (32).

Several studies have reported the increase in rates of antidepressant prescribing in the general population across the UK, which our findings mirror. (33-35) In Scotland the number of antidepressant prescriptions rose from 1.16 to 3.53 million per year between 1992 and 2006, (36) and women were prescribed antidepressants more frequently than men. (33) The reasons for the increase are unclear and have been attributed to multiple factors such as the availability of newer classes of drugs with fewer side effects, improved management of depression, lack of availability of alternative interventions, (36) a widening of clinical uses (33) and patient expectations. Earlier studies have cited concerns that depression may have been underdiagnosed in the population with intellectual disabilities (37). One American study which retrospectively analysed outpatient psychiatric charts reported a higher than expected rate of antidepressant prescribing for the subgroup with intellectual disabilities and suggested this was indicative of increasing diagnosis of depressive disorders in adults with intellectual disabilities (38). Another US study analysed data from adults with intellectual disabilities living in community settings in New York State between 2006 and 2007 also reported a higher than expected rate of antidressant prescribing in this group (39). The substantial increase in antidepressant prescribing observed in the current study may indicate improved diagnosis in primary care for this population. (24) This study has also observed that problem behaviours were independently associated with antidepressant prescribing in adults with intellectual disabilities. However a systematic review of antidepressants and problem behaviours management in people with intellectual disabilities concluded that evidence of their effectiveness in this context is lacking (40). Longitudinal patterns of antidepressant prescribing require further investigation.

Strengths and limitation

For peer review only - http://bmjopen.bmj.com/site/about/guidelines.xhtml

Strengths of the study include its large size, the longitudinal design, the detailed ascertainment of the population with intellectual disabilities, and the detailed health assessments at T1. The crosssectional cohorts were population-based at T1 and T2, so representative more widely of the population with intellectual disabilities. Additionally, the linked cohort was similar in characteristics with the whole cohort at T1, also suggesting it is representative and hence that the results are generalisable. The period of 12 weeks extraction of PIS data was selected to account for the frequency of prescriptions being issued. It included both regular and as-required drugs; given the 12 week prescribing period it is likely that the as-required medication was being actively used (as a fresh prescription had been issued and was encashed by the person with intellectual disabilities/their carer during this period). The time period for encashment was identical at both time points for the longitudinal, linked cohort. As a matter of caution in interpreting the data, the case-conferenced clinical mental ill-health diagnoses agreed by the study psychiatrists were used rather than ICD-10 or DSM-IV-TR diagnoses, in view of the under-recording of mental ill-health that these two classification systems produce with this population: had we used either of these classifications, our results would have been even more striking in terms of the discrepancy between mental ill-health and prescription of antipsychotics.

Only 73% of general practices agreed to data extraction, and this combined with deaths are likely to be the main reasons for 545/1,190 of the participants being linked in the T2 data, 10 years later. Limitations are the different methods of data collection, with specialist individual assessments at T1 and electronic data extraction at T2; in particular there is a large proportion of missing information and there may be inaccuracies on recorded level of intellectual disabilities in the general practitioner data at T2, so comparison of this variable between the T1 and T2 cohorts is limited. Additionally, there is lack of mental ill-health data at T2. The study did not investigate changes in dosages, polypharmacy, or duration of use. Some antipsychotic drugs are licenced for indications other than psychosis, and it is possible that other conditions accounted for their use e.g. promazine, whereas antidepressants and antiepileptics have seen increased use in the general population over this time period for neuralgic pain. We do not know how relevant this is to people with intellectual disabilities who may have difficulties in communicating pain, and note that encashed antiepileptics did not increase between the two cohorts.

Implications for research and practice

For peer review only - http://bmjopen.bmj.com/site/about/guidelines.xhtml

This study has demonstrated that fewer new antipsychotic prescriptions are being initiated, but those prescribed antipsychotics in T1 were unlikely to have these drugs withdrawn over the next decade demonstrating possible reluctance from carers, families and individuals combined with a lack of evidence available to prescribers to direct cessation interventions (22, 41) . The issue therefore remains far from addressed, and the risks of long-term health problems, death, and impact on quality of life associated with long-term antipsychotic prescriptions still needs further highlighting. (42) This study reinforces the need for frequent medication reviews for people with intellectual disabilities, alongside further research to investigate the long-term effects of antipsychotic medications on this population. (8) Further research to examine the barriers to antipsychotic drug reduction and to evaluate approaches to promoting reduction and withdrawal of antipsychotics for people with intellectual disabilities is needed. There is a dearth of evidence on antidepressant prescribing in the population with intellectual disabilities. The sharp increase in antidepressant prescribing observed in this study demands further research to understand the drivers for this. The association between increasing age and prescribing of antipsychotics and antidepressants also supports calls for research to investigate the implications of long-term psychotropic prescribing on older people with intellectual disabilities.(43)

Competing interests

All authors have completed the ICMJE uniform disclosure form at www.icmje.org/coi_disclosure.pdf and declare: no support from any organisation for the submitted work; no financial relationships with any organisations that might have an interest in the submitted work in the previous three years; no other relationships or activities that could appear to have influenced the submitted work .

Author statement

AH and PMcS analysed the data, jointly interpreted it, AH wrote the first draft of the manuscript, AH, S-AC, DK, CM, and LA jointly conceived the project, interpreted the data and contributed to the manuscript. AMcI contributed to additional analyses in response to reviewer comments. All authors approved the final version of the manuscript. S-AC is the study guarantor.

For peer review only - http://bmjopen.bmj.com/site/about/guidelines.xhtml

Funding

The study was funded by the Greater Glasgow Health Board, the Scottish Government via the Scottish Learning Disabilities Observatory and from the UK Medical Research Council (grant number: MC_PC_1717).

The study sponsors and funders had no role in the study design; in the collection, analysis, and interpretation of data; in the writing of the report; and in the decision to submit the article for publication.

The researchers are independent from the funders.

For peer review only

Statement

All authors had full access to all of the data (including statistical reports and tables) in the study and can take responsibility for the integrity of the data and the accuracy of the data analysis.

Transparency declaration

The manuscript's guarantor (S-AC) affirms that the manuscript is an honest, accurate, and transparent account of the study being reported; that no important aspects of the study have been omitted; and that there were no discrepancies from the study as planned.

Data sharing

No additional data is available

Acknowledgements

We are grateful to all the participants and their carers, to NHS Greater Glasgow learning disabilities primary care liaison team, to NHS Greater Glasgow and Clyde Safe Haven. This work uses data provided by patients and collected by the NHS as part of their care and support.

For peer review only - http://bmjopen.bmj.com/site/about/guidelines.xhtml

REFERENCES

1. Cooper SA, Smiley E, Morrison J, Williamson A, Allan L. Mental ill-health in adults with intellectual disabilities: prevalence and associated factors. Br J Psychiatry. 2007;190:27-35.
2. Cooper SA, Smiley E, Morrison J, Allan L, Williamson A, Finlayson J, et al. Psychosis and adults with intellectual disabilities - Prevalence, incidence, and related factors. Soc Psych Psych Epid. 2007;42(7):530-6.
3. Lunsky Y, Khuu W, Tadrous M, Vigod S, Cobigo V, Gomes T. Antipsychotic Use With and Without Comorbid Psychiatric Diagnosis Among Adults with Intellectual and Developmental Disabilities. Can J Psychiatry. 2017:706743717727240.
4. Lunsky Y, Khuu W, Tadrous M, Vigod S, Cobigo V, Gomes T. Antipsychotic Use With and Without Comorbid Psychiatric Diagnosis Among Adults with Intellectual and Developmental Disabilities. Can J Psychiat. 2018;63(6):361-9.
5. Glover G, Williams R. Prescribing of psychotropic drugs to people with learning disabilities and/or autism by general practitioners in England. London: Public Health England; 2015.
6. Kuijper Gd, Hoekstra P, Visser F, Scholte FA, Penning C, Evenhuis H. Use of antipsychotic drugs in individuals with intellectual disability (ID) in the Netherlands: prevalence and reasons for prescription. Journal of Intellectual Disability Research. 2010;54(7):659-67.
7. NICE. Challenging Behaviour and Learning Disabilities: Prevention and interventions for people with learning disabilities whose behaviour challenge. London: NICE; 2015.
8. Matson JL, Mahan S. Antipsychotic drug side effects for persons with intellectual disability. Res Dev Disabil. 2010;31(6):1570-6.
9. Doan TN, Lennox NG, Taylor-Gomez M, Ware RS. Medication use among Australian adults with intellectual disability in primary healthcare settings: A cross-sectional study. J Intellect Dev Dis. 2013;38(2):177-81.
10. Sheehan R, Hassiotis A, Walters K, Osborn D, Strydom A, Horsfall L. Mental illness, challenging behaviour, and psychotropic drug prescribing in people with intellectual disability: UK population based cohort study. BMJ. 2015;351:h4326.
11. Glassman AH, Bigger JT. Antipsychotic drugs: Prolonged QTc interval, torsade de pointes, and sudden death. Am J Psychiat. 2001;158(11):1774-82.
12. Cornett EM, Novitch M, Kaye AD, Kata V, Kaye AM. Medication-Induced Tardive Dyskinesia: A Review and Update. Ochsner J. 2017;17(2):162-72.
13. Stroup TS, Gray N. Management of common adverse effects of antipsychotic medications. World Psychiatry. 2018;17(3):341-56.
14. Sultan RS, Wang S, Crystal S, Olfson M. Antipsychotic Treatment Among Youths With Attention-Deficit/Hyperactivity Disorder. Jama Netw Open. 2019;2(7).
15. Olfson M, King M, Schoenbaum M. Treatment of Young People With Antipsychotic Medications in the United States. Jama Psychiat. 2015;72(9):867-74.
16. Sultan RS, Correll CU, Schoenbaum M, King M, Walkup JT, Olfson M. National Patterns of Commonly Prescribed Psychotropic Medications to Young People. J Child Adol Psychop. 2018;28(3):158-65.
17. Tyrer P, Cooper SA, Hassiotis A. Drug treatments in people with intellectual disability and challenging behaviour. BMJ. 2014;349:g4323.
18. NICE. Mental health problems in people with learning disabilities: prevention, assessment and management. London: NICE; 2016.
19. Health Do. Transforming care: A national response to Winterbourne View Hospital:

Department of Health Review Final Report. 2012.
20. Disability FoPoI. Psychotropic drug prescribing for people with intellectual disability, mental health problems and/or behaviours that challenge: practice guidelines. London: The Royal College of Psychiatrists; 2016.
21. England N. Stopping over medication of people with a learning disability, autism or both (STOMP) 2019 [29/01/19]. Available from: https://www.england.nhs.uk/learning-disabilities/improving-health/stomp/.

For peer review only - http://bmjopen.bmj.com/site/about/guidelines.xhtml

22. Song M, Ware R, Doan TN, Harley D. Psychotropic medication use in adults with intellectual disability in Queensland, Australia, from 1999 to 2015: a cohort study. J Intellect Disabil Res. 2019.
23. Sparrow SS, Cicchetti DV. Diagnostic Uses of the Vineland Adaptive-Behavior Scales. J Pediatr Psychol. 1985;10(2):215-25.
24. Organization WH. The ICD-10 Classification of Mental and Behavioral Disorders: Diagnostic Criteria for Research. Geneva, Switzerland: World Health Organization; 1993.
25. Association AP. Diagnostic and Statistical Manual of Mental Disorders (4th edn, text revision) (DSM-IV-TR). Washington DC2000.
26. Szymanski LS. DC-LD (Diagnostic criteria for psychiatric disorders for use with adults with learning Disabilities Mental retardation). Journal of Intellectual Disability Research. 2002;46:525-7.
27. Alvarez-Madrazo S, McTaggart S, Nangle C, Nicholson E, Bennie M. Data Resource Profile: The Scottish National Prescribing Information System (PIS). International Journal of Epidemiology. 2016;45(3):714-5f.
28. Sheehan R, Hassiotis A, Walters K, Osborn D, Strydom A, Horsfall L. Mental illness, challenging behaviour, and psychotropic drug prescribing in people with intellectual disability: UK population based cohort study. Bmj-Brit Med J. 2015;351.
29. O'Dwyer C, McCallion P, Henman M, McCarron M, O'Leary E, Burke E, et al. Prevalence and patterns of antipsychotic use and their associations with mental health and problem behaviours among older adults with intellectual disabilities. J Appl Res Intellect Disabil. 2019;32(4):981-93.
30. Deb S, Fraser W. The Use of Psychotropic Medication in People with Learning-Disability - Towards Rational Prescribing. Hum Psychopharm Clin. 1994;9(4):259-72.
31. Bowring DL, Totsika V, Hastings RP, Toogood S, McMahon M. Prevalence of psychotropic medication use and association with challenging behaviour in adults with an intellectual disability. A total population study. J Intellect Disabil Res. 2017;61(6):604-17.
32. Axmon A, El Mrayyan N, Eberhard J, Ahlstrom G. Pharmacotherapy for mood and anxiety disorders in older people with intellectual disability in comparison with the general population. BMC Psychiatry. 2019;19(1):238.
33. Lockhart P, Guthrie B. Trends in primary care antidepressant prescribing 1995-2007: a longitudinal population database analysis. Br J Gen Pract. 2011;61(590):e565-72.
34. Munoz-Arroyo R, Sutton M, Morrison J. Exploring potential explanations for the increase in antidepressant prescribing in Scotland using secondary analyses of routine data. Br J Gen Pract. 2006;56(527):423-8.
35. Hollinghurst S, Kessler D, Peters TJ, Gunnell D. Opportunity cost of antidepressant prescribing in England: analysis of routine data. Brit Med J. 2005;330(7498):999-1000.
36. Morrison J, Anderson MJ, Sutton M, Munoz-Arroyo R, McDonald S, Maxwell M, et al. Factors influencing variation in prescribing of antidepressants by general practices in Scotland. Br J Gen Pract. 2009;59(559):e25-31.
37. Robertson J, Emerson E, Gregory N, Hatton C, Kessissoglou S, Hallam A. Receipt of psychotropic medication by people with intellectual disability in residential settings. Journal of Intellectual Disability Research. 2000;44:666-76.
38. Hurley AD, Folstein M, Lam N. Patients with and without intellectual disability seeking outpatient psychiatric services: diagnoses and prescribing pattern. J Intellect Disabil Res. 2003;47(Pt 1):39-50.
39. Tsiouris JA, Kim SY, Brown WT, Pettinger J, Cohen IL. Prevalence of Psychotropic Drug Use in Adults with Intellectual Disability: Positive and Negative Findings from a Large Scale Study. Journal of Autism and Developmental Disorders. 2013;43(3):719-31.
40. Sohanpal SK, Deb S, Thomas C, Soni R, Lenotre L, Unwin G. The effectiveness of antidepressant medication in the management of behaviour problems in adults with intellectual disabilities: a systematic review. J Intellect Disabil Res. 2007;51(Pt 10):750-65.
41. McNamara R, Randell E, Gillespie D, Wood F, Felce D, Romeo R, et al. A pilot randomised controlled trial of community-led ANtipsychotic Drug REduction for Adults with Learning Disabilities. Health Technol Assess. 2017;21(47):1-92.
42. Scheifes A, Walraven S, Stolker JJ, Nijman HL, Egberts TC, Heerdink ER. Adverse events and the relation with quality of life in adults with intellectual disability and challenging behaviour using psychotropic drugs. Res Dev Disabil. 2016;49-50:13-21.

For peer review only - http://bmjopen.bmj.com/site/about/guidelines.xhtml

43. O'Dwyer M, Peklar J, Mulryan N, McCallion P, McCarron M, Henman MC. Prevalence, patterns and factors associated with psychotropic use in older adults with intellectual disabilities in Ireland. Journal of Intellectual Disability Research. 2017;61(10):969-83.

Figure legends

Figure 1. Participant flow diagram

Figure 2. Types of mental ill-health experienced by people prescribed antipsychotics at T1 n=292

For peer review only

For peer review only - http://bmjopen.bmj.com/site/about/guidelines.xhtml

Figure 1. Participant flow diagram

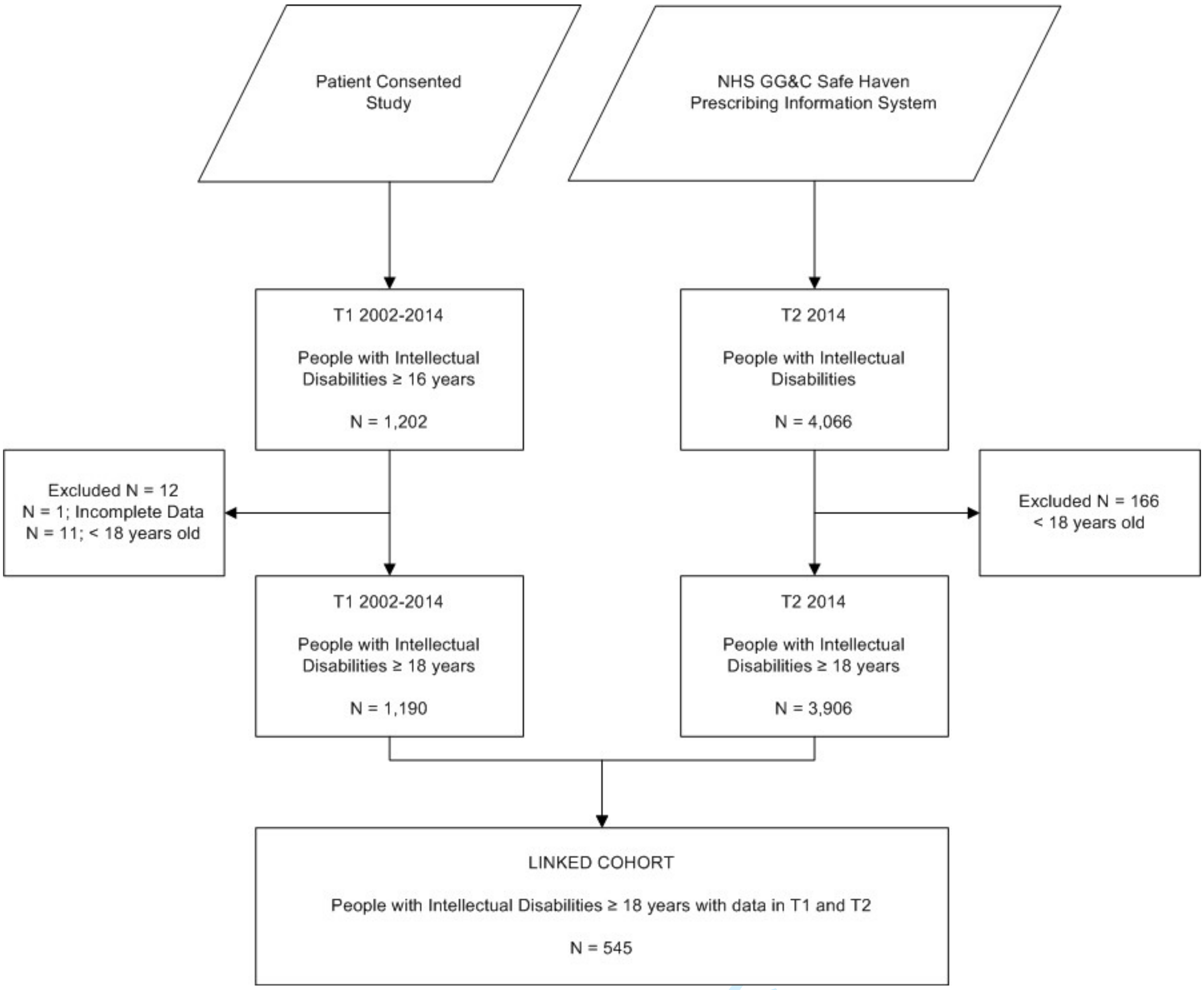

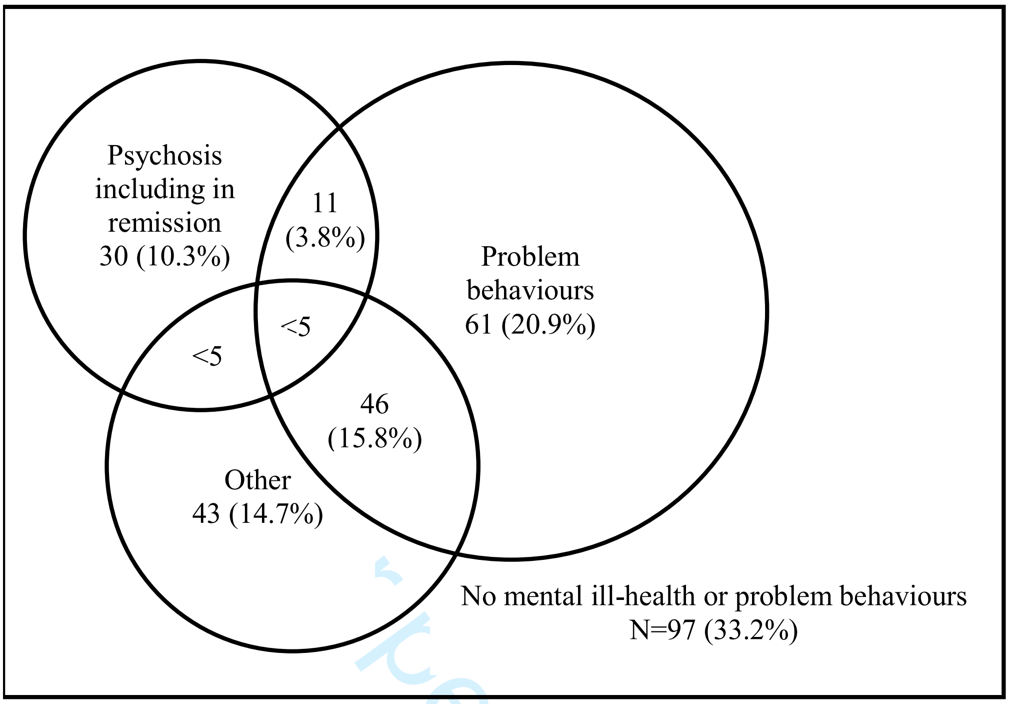

Figure 2. Types of mental ill-health experienced by people prescribed antipsychotics at T1 n=292

For peer review only

Supplementary table: Multivariable analysis of factors at T1 associated with psychotropic prescriptions at T2 for the linked cohort (N=545)

	Antipsychotics		Antidepressants		Hypnotics/anxiolytics		Antiepileptics		Lithium*	
	OR (95% CI)	p-value	OR (95% CI)	p-value	OR (95% CI)	p-value	OR (95% CI)	p-value	OR (95% CI)	p-value
Male sex	0.96 (0.63, 1.47)	0.848	0.59 (0.38, 0.89)	0.013	0.83 (0.46, 1.50)	0.545	0.97 (0.66, 1.42)	0.874	0.92 (0.25, 1.09)	0.894
Age at T1	1.04 (1.02, 1.06)	<0.001	1.01 (1.00, 1.03)	0.167	1.00 (0.98, 1.02)	0.993	0.99 (0.98, 1.00)	0.158	1.04 (0.99, 1.09)	0.160
Level of ID	-	0.018	-	0.002	-	0.473	-	< 0.001	-	0.464
Mild	REF		REF		REF		REF		REF	
Moderate	1.71 (1.02, 2.87)	0.041	0.90 (0.55, 1.48)	0.680	1.16 (0.56, 2.38)	0.690	1.56 (0.99, 2.48)	0.057	2.91 (0.68, 12.53)	0.152
Severe	2.31 (1.29, 4.15)	0.005	0.57 (0.30, 1.08)	0.083	0.75 (0.28, 1.97)	0.554	1.66 (0.96, 2.86)	0.071	0.90 (0.09, 8.80)	0.928
Profound	1.07 (0.53, 2.18)	0.853	0.22 (0.09, 0.56)	0.001	1.71 (0.71, 4.11)	0.229	3.57 (1.96, 6.5)	0.001	1.39 (0.14, 13.74)	0.780
Mental ill-health**	3.50 (2.02, 6.06)	< 0.001	2.73 (1.58, 4.70)	< 0.001	2.79 (1.34, 5.81)	0.006	1.33 (0.78, 2.27)	0.294	-	-
Problem behaviours	5.47 (3.25, 9.22)	< 0.001	2.75 (1.59, 4.76)	< 0.001	2.08 (1.00, 4.34)	0.050	1.35 (0.83, 2.17)	0.225	-	-

*Mental illness and problem behaviours excluded from Lithium model due to small numbers **Not including problem behaviours

For peer review only

STROBE (Strengthening The Reporting of OBservational Studies in Epidemiology) Checklist

A checklist of items that should be included in reports of observational studies. You must report the page number in your manuscript where you consider each of the items listed in this checklist. If you have not included this information, either revise your manuscript accordingly before submitting or note N/A.

Note: An Explanation and Elaboration article discusses each checklist item and gives methodological background and published examples of transparent reporting. The STROBE checklist is best used in conjunction with this article (freely available on the Web sites of PLoS Medicine at http://www.plosmedicine.org/, Annals of Internal Medicine at http://www.annals.org/, and Epidemiology at http://www.epidem.com/). Information on the STROBE Initiative is available at www.strobe-statement.org.

Section and Item	Item No.	Recommendation	Reported on Page No.
**Title and Abstract**	1	(*a*) Indicate the study's design with a commonly used term in the title or the abstract	
		(*b*) Provide in the abstract an informative and balanced summary of what was done and what was found	
**Introduction**			
Background/Rationale	2	Explain the scientific background and rationale for the investigation being reported	
Objectives	3	State specific objectives, including any prespecified hypotheses	
**Methods**			
Study Design	4	Present key elements of study design early in the paper	
Setting	5	Describe the setting, locations, and relevant dates, including periods of recruitment, exposure, follow-up, and data collection	
Participants	6	(*a*) *Cohort study*—Give the eligibility criteria, and the sources and methods of selection of participants. Describe methods of follow-up<br><br>*Case-control study*—Give the eligibility criteria, and the sources and methods of case ascertainment and control selection. Give the rationale for the choice of cases and controls<br><br>*Cross-sectional study*—Give the eligibility criteria, and the sources and methods of selection of participants	
		(*b*) *Cohort study*—For matched studies, give matching criteria and number of exposed and unexposed<br><br>*Case-control study*—For matched studies, give matching criteria and the number of controls per case	
Variables	7	Clearly define all outcomes, exposures, predictors, potential confounders, and effect modifiers. Give diagnostic criteria, if applicable	

For peer review only

Section and Item	Item No.	Recommendation	Reported on Page No.
Data Sources/ Measurement	8*	For each variable of interest, give sources of data and details of methods of assessment (measurement). Describe comparability of assessment methods if there is more than one group	
Bias	9	Describe any efforts to address potential sources of bias	
Study Size	10	Explain how the study size was arrived at	
Quantitative Variables	11	Explain how quantitative variables were handled in the analyses. If applicable, describe which groupings were chosen and why	
Statistical Methods	12	(*a*) Describe all statistical methods, including those used to control for confounding	
		(*b*) Describe any methods used to examine subgroups and interactions	
		(*c*) Explain how missing data were addressed	
		(*d*) *Cohort study*—If applicable, explain how loss to follow-up was addressed<br>*Case-control study*—If applicable, explain how matching of cases and controls was addressed<br>*Cross-sectional study*—If applicable, describe analytical methods taking account of sampling strategy	
		(*e*) Describe any sensitivity analyses	
**Results**			
Participants	13*	(a) Report numbers of individuals at each stage of study—eg numbers potentially eligible, examined for eligibility, confirmed eligible, included in the study, completing follow-up, and analysed	
		(b) Give reasons for non-participation at each stage	
		(c) Consider use of a flow diagram	
Descriptive Data	14*	(a) Give characteristics of study participants (eg demographic, clinical, social) and information on exposures and potential confounders	
		(b) Indicate number of participants with missing data for each variable of interest	
		(c) *Cohort study*—Summarise follow-up time (eg, average and total amount)	
Outcome Data	15*	*Cohort study*—Report numbers of outcome events or summary measures over time	
		Case-control study—Report numbers in each exposure category, or summary measures of exposure	
		Cross-sectional study—Report numbers of outcome events or summary measures	

For peer review only

Section and Item	Item No.	Recommendation	Reported on Page No.
Main Results	16	(*a*) Give unadjusted estimates and, if applicable, confounder-adjusted estimates and their precision (eg, 95% confidence interval). Make clear which confounders were adjusted for and why they were included	
		(*b*) Report category boundaries when continuous variables were categorized	
		(*c*) If relevant, consider translating estimates of relative risk into absolute risk for a meaningful time period	
Other Analyses	17	Report other analyses done—eg analyses of subgroups and interactions, and sensitivity analyses	
**Discussion**			
Key Results	18	Summarise key results with reference to study objectives	
Limitations	19	Discuss limitations of the study, taking into account sources of potential bias or imprecision. Discuss both direction and magnitude of any potential bias	
Interpretation	20	Give a cautious overall interpretation of results considering objectives, limitations, multiplicity of analyses, results from similar studies, and other relevant evidence	
Generalisability	21	Discuss the generalisability (external validity) of the study results	
**Other Information**			
Funding	22	Give the source of funding and the role of the funders for the present study and, if applicable, for the original study on which the present article is based	

*Give information separately for cases and controls in case-control studies and, if applicable, for exposed and unexposed groups in cohort and cross-sectional studies.

Once you have completed this checklist, please save a copy and upload it as part of your submission. DO NOT include this checklist as part of the main manuscript document. It must be uploaded as a separate file.

For peer review only

BMJ Open

Changes over a decade in psychotropic prescribing for people with intellectual disabilities: prospective cohort study

Journal:	*BMJ Open*
Manuscript ID	bmjopen-2020-036862.R2
Article Type:	Original research
Date Submitted by the Author:	08-Jul-2020
Complete List of Authors:	Henderson, Angela; University of Glasgow, Institute of Health and Wellbeing<br>Mcskimming, Paula; University of Glasgow, Robertson Centre for Biostatistics<br>Kinnear, Deborah; University of Glasgow, Institute of Health and Wellbeing<br>McCowan, Colin ; University of Saint Andrews, School of Medicine<br>McIntosh, Alasdair; University of Glasgow, Robertson Centre for Biostatistics<br>Allan, Linda; University of Glasgow, Institute of Health and Wellbeing<br>Cooper, Sally-Ann; University of Glasgow, Institute of Health and Wellbeing
**Primary Subject Heading**:	Mental health
Secondary Subject Heading:	Pharmacology and therapeutics
Keywords:	MENTAL HEALTH, PSYCHIATRY, CLINICAL PHARMACOLOGY, Adult psychiatry < PSYCHIATRY, Depression & mood disorders < PSYCHIATRY, Schizophrenia & psychotic disorders < PSYCHIATRY

SCHOLARONE™
Manuscripts

I, the Submitting Author has the right to grant and does grant on behalf of all authors of the Work (as defined in the below author licence), an exclusive licence and/or a non-exclusive licence for contributions from authors who are: i) UK Crown employees; ii) where BMJ has agreed a CC-BY licence shall apply, and/or iii) in accordance with the terms applicable for US Federal Government officers or employees acting as part of their official duties; on a worldwide, perpetual, irrevocable, royalty-free basis to BMJ Publishing Group Ltd ("BMJ") its licensees and where the relevant Journal is co-owned by BMJ to the co-owners of the Journal, to publish the Work in this journal and any other BMJ products and to exploit all rights, as set out in our licence.

The Submitting Author accepts and understands that any supply made under these terms is made by BMJ to the Submitting Author unless you are acting as an employee on behalf of your employer or a postgraduate student of an affiliated institution which is paying any applicable article publishing charge ("APC") for Open Access articles. Where the Submitting Author wishes to make the Work available on an Open Access basis (and intends to pay the relevant APC), the terms of reuse of such Open Access shall be governed by a Creative Commons licence – details of these licences and which Creative Commons licence will apply to this Work are set out in our licence referred to above.

Other than as permitted in any relevant BMJ Author's Self Archiving Policies, I confirm this Work has not been accepted for publication elsewhere, is not being considered for publication elsewhere and does not duplicate material already published. I confirm all authors consent to publication of this Work and authorise the granting of this licence.

For peer review only

Title: Changes over a decade in psychotropic prescribing for people with intellectual disabilities: prospective cohort study

Authors: Angela Henderson[1], Deputy Director, Scottish Learning Disabilities Observatory
Paula McSkimming[2], Biostatistician
Deborah Kinnear[1], Senior Lecturer
Colin McCowan[3], Professor in Health Data Science
Alasdair McIntosh[2], Junior Biostatistician
Linda Allan[1], Honorary Clinical Associate Professor
*Sally-Ann Cooper[1], Professor of Learning Disabilities

*Correspondence. Sally-Ann.Cooper@Glasgow.ac.uk 0141 211 0690

1. Institute of Health and Wellbeing, University of Glasgow, Mental Health and Wellbeing research group, 1st Floor, Administrative Building, Gartnavel Royal Hospital, 1055 Great Western Road, Glasgow, G12 0XH
2. Robertson Centre for Biostatistics, Institute of Health and Wellbeing, University of Glasgow, Boyd Orr Building, University Avenue, Glasgow, G12 8QQ
3. School of Medicine, University of St Andrews, North Haugh, St Andrews KY16 9TF

Word count: 4,249

For peer review only

ABSTRACT

OBJECTIVES: To investigate psychotropic prescribing in the intellectual disabilities population over 10 years, and associated mental ill-health diagnoses.

DESIGN: (a) Comparison of cross-sectional data in 2002-2004 (T1), and 2014 (T2). (b) Longitudinal cohort study with detailed health assessments at T1, and record linkage to T2 prescribing data.

SETTING: General community.

PARTICIPANTS: (a) 1,190 adults with intellectual disabilities in T1 compared with 3,906 adults with intellectual disabilities in T2. (b) 545/1,190 adults with intellectual disabilities in T1 were alive and their records linked to T2 prescribing data.

MAIN OUTCOME MEASURES: Encashed regular and as-required psychotropic prescriptions.

RESULTS: (a) 50.7% (603/1,190) in T1, and 48.2% (1,881/3,906) in T2 were prescribed 1+ psychotropics: antipsychotics 24.5% (292/1,190) in T1 and 16.7% (653/3,906) in T2, antidepressants 11.2% (133/1,190) in T1 and 19.1% (746/3,906) in T2. 30.0% (62/292) prescribed antipsychotics in T1 had psychosis or bipolar disorder, 33.2% (97/292) had no mental ill-health or problem behaviours, 20.6% (60/292) had problem behaviours but no psychosis/bipolar disorder. (b) Psychotropics increased from 47.0% (256/545) in T1 to 57.8% (315/545) in T2 (p<0.001): antipsychotics did not change (OR=1.18; CI (0.87, 1.60); p=0.280), there was an increase for antidepressants (OR=2.80; CI 1.96, 4.00; p<0.001), hypnotics/anxiolytics (OR=2.19; CI 1.34, 3.61; p=0.002), and antiepileptics (OR=1.40; CI 1.06, 1.84; p=0.017). Antipsychotic prescribing increased for people with problem behaviours in T1 (OR=6.45, CI 4.41, 9.45; p<0.001), more so than for people with other mental ill-health in T1 (OR 4.11, CI 2.76, 6.11; p<0.001).

CONCLUSIONS: Despite concerns about antipsychotic prescribing and guidelines recommending their withdrawal, it appears that whilst fewer antipsychotic prescriptions were initiated by T2 than in T1, people were not withdrawn from them once commenced. People with problem behaviours had increased prescribing. There was also a striking increase in antidepressant prescriptions. Adults with intellectual disabilities need frequent and careful medication reviews.

Key words: intellectual disabilities, psychotropics, antipsychotics, antidepressants, hypnotics, anxiolytics, anti-epileptics, lithium, mental ill-health

For peer review only - http://bmjopen.bmj.com/site/about/guidelines.xhtml

Article summary

Strengths and limitations

The strengths of this study are:

- The large cohort size, longitudinal design, detailed ascertainment of the population with intellectual disabilities, and the detailed health assessments at T1.
- The cross-sectional cohorts were population-based at T1 and T2, and representative of the population with intellectual disabilities; the linked cohort was similar in characteristics to the cross-sectional cohort at T1, suggesting it is also representative and therefore that the results are generalisable.

The limitations of this study are:

- Only 73% of general practices agreed to data extraction, and this combined with deaths are likely to be the main reasons for 545/1,190 of the participants being linked in the T2 data, 10 years later.
- The different methods of data collection, with specialist individual assessments at T1 and electronic data extraction at T2; in particular there is a large proportion of missing information and may be inaccuracies on recorded level of intellectual disabilities in the general practitioner data at T2 limiting comparability of this variable between the T1 and T2 cohorts.
- The study did not investigate changes in dosages, polypharmacy or duration of use and there is a lack of mental ill-health data at T2.

INTRODUCTION

Mental ill-health is common in people with intellectual disabilities. (1) The prevalence of psychosis in this population is reported to be around 4% based on cross-sectional data, and the rate of people with a first psychotic episode is about 10 times that of the general population. (2) Whilst the rates of psychosis are relatively high, antipsychotics are often prescribed for adults with intellectual disabilities who do not have a record of severe mental ill-health (3, 4), often for problem behaviours, (5-9) and despite limited evidence to support their use beyond short-term sedation. (7) Indeed, 71% of people with intellectual disabilities who are prescribed antipsychotics have been reported to have no record of serious mental ill-health. (10) This is important, as antipsychotics have numerous disabling, painful, and disfiguring side effects, some of which are life threatening such as tardive dyskinesia, cardiac arrhythmias, and sudden cardiac death. (11-13) Antipsychotics are also frequently prescribed

For peer review only - http://bmjopen.bmj.com/site/about/guidelines.xhtml

for children and young people with a range of developmental disabilities and problem behaviours (14, 15), and in the young general population, rates increase during adolescence. (16)

Concerns have repeatedly been raised about the overuse of antipsychotics, and the need for more proportionate prescribing for people with intellectual disabilities. (7, 17-19) In 2016 a national campaign was launched by NHS England in partnership with the Royal Colleges of General Practitioners, Psychiatrists, and Nursing, the Royal Pharmaceutical Society, and British Psychological Society to address these concerns in England: "Stopping over medication of people with a learning disability, autism or both (STOMP)". Guidelines from STOMP, the National Institute for Health and Care Excellence, and the Royal College of Psychiatrists highlight that prescribers, where appropriate, should reduce or withdraw antipsychotics for people with intellectual disabilities who do not have psychosis. (7, 20, 21) However, there is very little empirical evidence from the UK on any changes in antipsychotic prescribing patterns over time. An exception is a study by Sheehan et al (2015) that extracted data from general practice records on 33,016 adults with a record of intellectual disabilities, with a median follow-up of 5.5 years. (10) They reported the incidence of new psychotropic prescription to be 518/10,000 person years. Prescriptions of antipsychotics fell by 4% per year over the study period, as did mood stabilisers, whilst there was no consistent trend for antidepressants or anxiolytics/hypnotics. Sheehan et al. (2015) reported that 47% of those with "challenging behaviour" had received antipsychotic drugs, but only 12% had a record of severe mental ill-health, and that 26% of those prescribed antipsychotics did not have a record of severe mental ill-health or "challenging behaviour". A limitation of this study is in the identification of 'challenging behaviour' through a heterogeneous list of 45 Read codes (the system used in general practices in the UK to code diagnoses). Read codes do not provide a robust method for ascertaining problem behaviours. Additionally incomplete and variable recording practices do not always accurately reflect a person's health. (10) Another study from Australia investigated psychotropic medication use between 1999 and 2015 in a cohort of 138 participants (22) and also found a strong association between problem behaviours and psychotropic medication. In this cohort the study reported that once psychotropic medications were prescribed they were unlikely to be removed, and observed little change in prescribing of antipsychotics between 1999 and 2015 (24/138 (24%) to 23/92 (23%)). A sharp increase in the prescribing of antidepressants from 16.7% to 36.1% across the same period was also observed. However, whilst this was a longitudinal cohort not all

For peer review only - http://bmjopen.bmj.com/site/about/guidelines.xhtml

participants took part in all waves of data collection, therefore it is not possible to ascertain within group changes in prescribing.

Aim

The aim of this study is to investigate changes over a decade in psychotropic prescribing for adults with intellectual disabilities, and the diagnoses associated with antipsychotics, from detailed psychiatric assessments.

METHODS

Ethical approval

NHS Greater Glasgow Primary Care Trust, Community & Mental Health Research Ethics Committee granted ethical approval (project number 01/44). Between 2002-2004 (T1), individual consent to participate was taken in line with Scottish law. In 2014 (T2), 191/263 (73%) general practices in NHS Greater Glasgow and Clyde area participated, and the NHS Greater Glasgow and Clyde Local Privacy Advisory Committee approved electronic extraction and linkage of primary care records.

Participants

In 2000-2001, a primary care intellectual disabilities register was established of adults with intellectual disabilities, aged ≥16 years, living in the NHS Greater Glasgow area. This was delivered through partnership between the intellectual disabilities clinical service and all general practitioners in the area. People with intellectual disabilities were identified through social work services for people with intellectual disabilities; local authority funding arrangements for people receiving paid support of any kind, including day opportunities; local specialist health services for people with intellectual disabilities; the Health Board; and general practices who were financially incentivised to identify their registered patients with intellectual disabilities (100% of general practices participated). Intellectual disabilities nurses reviewed all cases on the register to ascertain if intellectual disabilities were present, those that did not have intellectual disabilities were removed from the register. The register was then updated annually by the general practices and the intellectual disabilities clinical service. Between 2002-2004, the register was used to invite people living in a *representative part of the Health Board area* to partipate in the study, 67% agreed to take part: these participants were recruited to a longitudinal cohort between 2002 and 2004 (T1), and had detailed health assessments at that time. 1,190 were aged ≥18 years, and comprise the study

For peer review only - http://bmjopen.bmj.com/site/about/guidelines.xhtml

population reported here. In 2014 (T2), for people on the register and living in *the whole of the Health Board area,* data was extracted from primary care records; 73% of general practices in the Health Board agreed to the data extraction, data was extracted on their 3,906 patients with intellectual disabilities aged ≥18 years, who comprise the study population reported here.

Process and measures

Semi-structured individual health assessments, including medication review, assessment of level of intellectual disabilities (via structured questions on abilities, and the Vineland Scale (23)), mental ill-health symptoms including problem behaviours and autism, were conducted at T1 by one of six intellectual disabilities nurses and one of three general practitioners with a special interest in intellectual disabilities. These were preceeded by review and data collection from the person's general practitioner medical records, and then conducted with the person with intellectual disabilities and their carer(s). This included a review of drug charts for the participants in supported care. The 54% of individuals identified with possible, probable, or definite mental ill-health (including problem behaviours and autism) were then assessed by the study psychiatrists who were specialists in intellectual disabilities psychiatry. Information from each person's psychiatric assessment was reviewed by two psychiatrists who case-conferenced and agreed the classification of the mental ill-health, using ICD-10-DCR (24), DSM-IV-TR (25), DC-LD (26) and clinical criteria. Details have been previously reported. (1) Given that ICD-10 and DSM criteria function poorly for adults with moderate to severe intellectual disabilities (particularly with regards to problem behaviours), in this study we report the clinical diagnoses agreed together by the study psychiatrists. Data collection was over a two year period. Drugs were coded using British National Formulary (BNF) codes.

At T2, the 3,906 adults with intellectual disabilities identified from primary care records were record linked to Prescribing Information System (PIS) data, using the Community Health Index (CHI), which is the NHS patient identification number, unique to each person. PIS is Scotland's electronic record of all encashed prescriptions (i.e. not prescriptions issued, or drugs administered, but those that the carers/person with intellectual disabilities actually took to a pharmacist and exchanged for the drugs), and includes a record of the BNF code of each prescribed drug. (27) Prescribing information was then extracted using BNF codes for the 3,906 adults with intellectual disabilities to identify all prescriptions of antipsychotics,

For peer review only - http://bmjopen.bmj.com/site/about/guidelines.xhtml

antidepressants, antiepileptics, lithium, and hypnotics/anxiolytics across a specific 12-week prescribing window in 2014, including both regular prescriptions and as-required medication. To establish the longitudinal cohort the CHI number was used to identify T1 participants in the T2 dataset, enabling comparison of encashed medications across the decade. Only participants with complete data who were aged 18 years and over were included in the analyses (Figure 1).

Statistical analysis

Subject characteristics and prescribing information were summarised descriptively with mean and standard deviation (SD) for continuous outcomes and number and percentage for categorical outcomes at each time point (T1 and T2). Prescribing information at each time point was summarised using binary variables for each class of medications of interest (yes/no), allowing prescribing patterns to be investigated between the two time points in the study using McNemar's tests on the subset of the linked cohort, for whom there were prescribing records at both T1 and T2. This analysis was extended to explore whether there were associations between time or the subject characteristics at T1, with each prescription category using binary logistic regression models. Each model included multiple explanatory variables, specifically; time as a binary variable to indicate each time point T1 and T2; sex; age as a continuous measure; level of intellectual disabilities as four-level categorical variable; presence of mental ill-health (yes/no, excluding problem behaviours); having problem behaviours (yes/no); and a binary dependant variable for each class of medication (yes/no). Logistic regression models were also fitted with the above T1 subject characteristics to explore their association with each prescribing category specifically at T2. Odd ratios are reported for all logistic regression models with corresponding 95% confidence intervals (CIs) and p-values. A p-value of less than 0.05 is considered as statistically significant. Statistical analyses were conducted using SAS version 9.3.

Patient and Public Involvement

The Scottish Learning Disabilities Observatory has a steering committee which meets twice a year and provides strategic direction and oversight of all of the Observatory's research, including this project. The steering committee includes people with learning disabilities from 'People First', a national group of self-advocates with intellectual disabilities.

RESULTS

For peer review only - http://bmjopen.bmj.com/site/about/guidelines.xhtml

Participant characteristics of the cross-sectional cohorts

Data for those who had incomplete data at T1 and for those who were aged under 18 years at either time point were excluded from further analyses. Table 1 shows participant characteristics; age, sex, level of intellectual disabilities at T1 (n=1,190) and T2 (n=3,906), and mental ill-health and epilepsy diagnoses at T1. No mental health or epilepsy data were available at T2.

Table 1. Participant characteristics for the cross-sectional cohorts at T1 and T2

Characteristic	T1 aged 18+years (N=1,190)	T2 aged 18+ years (N=3,906)
**Age Mean (SD)**	44.6 (14.3)	45.4 (15.5)
**Sex N(%):**		
Male	671 (56.4)	2,260 (57.9)
Female	519 (43.6)	1,646 (42.1)
**Level of intellectual disabilities N(%):**		
Mild	451 (37.9)	1047 (26.8)
Moderate	319 (26.8)	859 (22.0)
Severe	233 (19.6)	595 (15.2)
Profound	187 (15.7)	197 (5.0)
Unknown	0	1,208 (30.9)
**Epilepsy N(%)**	419 (35.2%)	Not collected
**Type of mental ill-health N(%):**		
Psychosis, including psychosis in remission	52 (4.4)	Not collected
Problem behaviours	244 (20.5)	
Autism	80 (6.7)	
ADHD	15 (1.3)	
Unipolar depression	51 (4.3)	
Bipolar disorder	21 (1.8)	
Anxiety disorders	32 (2.7)	
Organic disorder	20 (1.7)	
Personality disorder	9 (0.8)	
Obsessive compulsive disorder	7 (0.6)	
Psychosexual disorder	< 5	
Other	15 (1.3)	
Mental ill-health (including problem behaviours)	438 (36.8)	
Mental ill-health (excluding problem behaviours)	194 (16.3)	

Prescribing for the cross-sectional cohorts

At least one psychotropic was prescribed at T1 for 50.7% (603/1,190), and at T2 for 48.2% (1,881/3,906) (table 2) of participants. Antipsychotics were prescribed to 24.5% (292/1,190) of participants at T1 and 16.7% (653/3,906) at T2. At T1, antidepressants were prescribed for

For peer review only - http://bmjopen.bmj.com/site/about/guidelines.xhtml

11.2% (133/1,190), and at T2 for 19.1% (746/3,906) of participants. Hypnotic/anxiolytic, lithium, and anti-epileptic prescribing was similar at T1 and T2.

The types of mental ill-health experienced by the 292 participants at T1 who were taking antipsychotics are shown in table 3. The most common diagnosis within this group was problem behaviours at 40.8% (119/292). Of note, 33.2% (97/292) of the people taking antipsychotics did not have any identified mental ill-health or problem behaviours. Figure 2 demonstrates the overlap between groups of the people who were taking antipsychotics at T1 and selected diagnoses.

Figure 2. Types of mental ill-health experienced by people prescribed antipsychotics at T1 n=292

Table 3 also shows the types of mental ill-health experienced by the 230 participants at T1 who were taking antipsychotics, after excluding people with psychosis (or psychosis in remission) or bipolar disorder (given that they would be expected to be prescribed antipsychotics, and given the considerable overlap between disorders shown in Figure 2). Most strikingly, 97/230 (42.2%) of those prescribed antipsychotics had no mental ill health or problem behaviours. The proportion of people in each diagnostic category, without co-occurring psychosis or bipolar disorder who were taking antipsychotics was considerable for all types: 11.7% (27/230) for autism, 7.0% (16/230) for unipolar depression, 2.6% (6/229) for anxiety disorders and 2.2% (5/230) or less for all other diagnoses.

Table 2. Psychotropics prescribed for the cross-sectional cohorts at T1 and T2

**Prescriptions**	**T1 aged 18+ years (N=1,190)**	**T2 aged 18+ years (N=3, 906)**
Any psychotropic drug	603 (50.7)	1,881 (48.2)
Antipsychotics	292 (24.5)	653 (16.7)
Antidepressants	133 (11.2)	746 (19.1)
Antiepileptics	333 (28.0)	1,028 (26.3)
Lithium	14 (1.2)	31 (0.8)
Hypnotics/anxiolytics	81 (6.8)	305 (7.8)
Missing data	0	3 (0.1)

Table 3. Types of mental ill-health at T1 experienced by people prescribed antipsychotics at T1, and after excluding people with psychosis and bipolar disorder

For peer review only - http://bmjopen.bmj.com/site/about/guidelines.xhtml

For peer review only

Mental ill-health at T1		Adults (18+ years) taking antipsychotics at T1	
**Diagnosis**	**All (N=1,190)**	**All (N=292)**	**Excluding people with psychosis and bipolar disorder (N=230)**
Psychosis, including psychosis in remission	52	45 (15.4)	-
Problem behaviours	244	119 (40.8)	100 (43.5)
Autism	80	30 (10.3)	27 (11.7)
ADHD	15	12 (4.1)	11 (4.8)
Unipolar depression	51	20 (6.9)	16 (7.0)
Bipolar disorder	21	17(5.8)	-
Anxiety disorders	32	7 (2.4)	6 (2.6)
Organic disorder	20	5 (1.7)	< 5
Personality disorder	9	5 (1.7)	< 5
Obsessive Compulsive Disorder	7	3 (1.0)	< 5
Psychosexual disorder	< 5	< 5	< 5
Other	15	6 (2.1)	5 (2.2)
Mental ill-health (including problem behaviours)	438	195 (66.8)	133 (57.8)
Mental ill-health (excluding problem behaviours)	194	76 (26.3)	33 (14.4)
No mental ill-health nor problem behaviours	752	97 (33.2)	97 (42.2)

- **Insert figure 2 here. Types of mental ill-health experienced by people prescribed antipsychotics at T1 -**

Participant characteristics of the longitudinal, linked cohort

The longitudinal, linked cohort included the 545 adults who were in the T1 cohort and who were also identified within the GP records at T2. Table 4 shows their age, sex, level of intellectual disabilities, epilepsy diagnosis, and mental ill-health at T1. They appear to be broadly representative of the whole cohort at T1 on these characteristics.

Table 4. Participant characteristics at T1 for people in the longitudinal cohort

Characteristic	T1 (aged 18+ years) N=545 (%)
**Age Mean (SD)**	41.8 (13.2)

For peer review only - http://bmjopen.bmj.com/site/about/guidelines.xhtml

**Sex N (%):**	
Male	322 (59.1)
Female	223 (40.9)
**Level of intellectual disabilities N (%):**	
Mild	237 (43.5)
Moderate	154 (28.3)
Severe	89 (16.3)
Profound	65 (11.9)
**Epilepsy**	173 (31.7)
**Type of mental ill-health N (%):**	
Psychosis, including psychosis in remission	32 (5.9)
Problem behaviours	109 (20.0)
Autism	38 (7.0)
ADHD	8 (1.5)
Unipolar depression	23 (4.2)
Bipolar disorder	10 (1.8)
Anxiety disorders	14 (2.6)
Organic disorder	< 5
Personality disorder	7 (1.3)
Obsessive compulsive disorder	< 5
Psychosexual disorder	<5
Other	6 (1.1)
Mental ill-health (including problem behaviours)	190 (34.9)
Mental ill-health (excluding problem behaviours)	81 (14.9)

Prescribing for the longitudinal, linked cohort

At least one psychotropic medication was prescribed at T1 for 47.0% (256/545), and for 57.8% (315/545) at T2 (table 5) which is a significant increase over time (p<0.001). Antidepressants were prescribed for 9.9% (54/545) of participants at T1, and at T2 for 22.0% (120/545), showing a significant increase (p<0.001). At T1, hypnotics/anxiolytics were prescribed for 4.6% (25/545) of participants, and at T2 for 9.4% (51/545), a significant increase (p<0.001). At T1, antiepileptics were prescribed for 24.8% (135/545) of participants, and at T2 for 31.0% (169/545), a significant increase (p<0.001). Prescribing patterns at T1 and T2 were similar for both antipsychotics and lithium. Of the 128 people prescribed antipsychotics at T1, 77.3% (99/128) were prescribed antipsychotics at T2; only 29 (22.7%) had been withdrawn, and 43/545 (7.9%) had started on an antipsychotic between the two timepoints.

Table 5. Psychotropic medications prescribed for the longitudinal, linked cohort at T1 and T2

Medication group	T1	T2	P-value

For peer review only - http://bmjopen.bmj.com/site/about/guidelines.xhtml

	(N=545)	(545)	
**Any psychotropic medication**	256 (47.0%)	315 (57.8%)	p<0.001
**Antipsychotics**	128 (23.5%)	142 (26.1%)	p=0.099
**Antidepressants**	54 (9.9%)	120 (22.0%)	p<0.001
**Hypnotics/anxiolytics**	25 (4.6%)	51 (9.4%)	P<0.001
**Antiepileptics**	135 (24.8%)	169 (31.0%)	P<0.001
**Lithium**	7 (1.3%)	10 (1.8%)	P=0.180

The logistic regression analyses, taking account of sex, age, level of intellectual disabilities, having mental ill-health (excluding problem behaviours) and having problem behaviours at T1 (Table 6) shows no significant difference in antipsychotic prescribing rate in the linked cohort over the decade (OR = 1.18; CI 0.87 to 1.60; p = 0.280), an increase in antidepressants (OR=2.80; CI 1.96 to 4.00; p<0.001), hypnotics/anxiolytics (OR=2.19; CI 1.34 to 3.6; p=0.002), and antiepileptic prescribing (OR=1.40; CI 1.06 to 1.84; p = 0.017). Sex was not independently associated with prescribing, except that women were more likely to have an increase in antidepressants than men after adjusting for time (OR = 0.53, CI 0.37 to 0.78; p<0.001). Older age had a small effect on prescribing for antipsychotics and antidepressants. Effects are also observed for level of intellectual disabilities. There was a gradient for antiepileptics (increased prescribing with increasing severity of intellectual disabilities) and a gradient for antidepressants (reduced prescribing with increasing severity of intellectual disabilities). However there was no gradient across different ability levels for antipsychotic prescribing. As expected, participants with a diagnosed mental health problem (excluding problem behaviours) at T1 were more likely to be prescribed antipsychotics (OR=4.11, CI 2.76 to 6.11; p < 0.001), antidepressants (OR=3.90, CI 2.53 to 6.02; p < 0.001), and hypnotics/anxiolytics (OR=3.25, CI 1.78 to 5.94; p < 0.001). Strikingly though, those with problem behaviours identified at T1 were over 6 times more likely to have increased prescribing of an antipsychotic (OR=6.45, CI 4.41 to 9.45; p < 0.001), over 3 times more likely for antidepressants (OR=3.44, CI 2.22 to 5.35; p < 0.001) and 3 times more likely for hypnotics/anxiolytics (OR=3.06, CI 1.72 to 5.44; p < 0.001).

The further regression (Supplementary table 1) investigating factors at T1 which are associated with prescribing at T2 (as opposed to change in prescribing reported in the

For peer review only - http://bmjopen.bmj.com/site/about/guidelines.xhtml

paragraph above) shows that women were more likely to be prescribed antidepressants at T2, that older age had a small effect for antipsychotics and antidepressants at T2, a gradient across ability level for antiepileptics, a relationship with moderate and severe (but not profound) intellectual disabilities for antipsychotics at T2, and less antidepressants for people with profound intellectual disabilities. Mental ill-health and problem behaviours at T1 predicted prescribing of all classes.

For peer review only

For peer review only - http://bmjopen.bmj.com/site/about/guidelines.xhtml

Table 6: Multivariable analysis of exploratory T1 factors and time with psychotropic prescriptions for the linked cohort (N=545)

	Antipsychotics		Antidepressants		Hypnotics/anxiolytics		Antiepileptics		Lithium*	
	OR (95% CI)	p-value	OR (95% CI)	p-value	OR (95% CI)	p-value	OR (95% CI)	p-value	OR (95% CI)	p-value
Time	1.18 (0.87, 1.60)	0.280	2.80 (1.96, 4.00)	< 0.001	2.19 (1.34, 3.60)	0.002	1.40 (1.06, 1.84)	0.017	0.69 (0.26, 1.84)	0.4612
Male sex	0.99 (0.73, 1.34)	0.954	0.53 (0.37, 0.76)	< 0.001	0.83 (0.52, 1.35)	0.456	1.02 (0.77, 1.35)	0.896	0.93 (0.34, 2.59)	0.890
Age at T1	1.04 (1.03, 1.05)	< 0.001	1.02 (1.00, 1.03)	0.010	1.01 (0.99, 1.03)	0.260	0.99 (0.98, 1.00)	0.057	0.96(0.93, 0.99)	0.016
Level of intellectual disabilities (compared with Mild ID)	-	< 0.001	-	0.001	-	0.444	-	< 0.001	-	0.094
Moderate	1.88 (1.30, 2.74)	< 0.001	0.82 (0.54, 1.24)	0.346	1.64 (0.92, 2.92)	0.093	1.78 (1.26, 2.51)	< 0.001	0.23(0.07, 0.71)	0.011
Severe	2.49 (1.61, 3.85)	< 0.001	0.62 (0.37, 1.03)	0.063	1.12 (0.55, 2.31)	0.750	2.30 (1.55, 3.41)	< 0.001	1.49(0.17, 13.36)	0.721
Profound	1.61 (0.97, 2.69)	0.067	0.22 (0.11, 0.46)	< 0.001	1.24 (0.58, 2.62)	0.579	4.73 (3.07, 7.31)	< 0.001	0.46 (0.08, 2.64)	0.386
Mental ill-health**	4.11 (2.76,	< 0.001	3.90 (2.53,	< 0.001	3.25 (1.78,	< 0.001	1.13 (0.76,	0.547	-	-

For peer review only - http://bmjopen.bmj.com/site/about/guidelines.xhtml

	6.11)		6.02)		5.94)		1.70)			
Problem behaviours	6.45 (4.41, 9.45)	< 0.001	3.44 (2.22, 5.35)	< 0.001	3.06 (1.72, 5.44)	< 0.001	1.27 (0.90, 1.81)	0.174	-	-

*Mental illness and problem behaviours excluded from Lithium model due to small numbers **Not including problem behaviours

For peer review only

For peer review only - http://bmjopen.bmj.com/site/about/guidelines.xhtml

DISCUSSION

Principal findings

Despite numerous calls and guidelines in the UK for the withdrawal of antipsychotic drugs from people with intellectual disabilities who do not have psychosis/bipolar disorders, (7, 20, 21) our longitudinal, linked cohort analysis demonstrates no progress over a decade. The comparison of the two cross-sectional cohorts does show a lower rate of antipsychotic prescribing in T2 than was observed in T1 but the rate is still high in T2, at 16.7% of the population. It appears that whilst few people are being withdrawn from antipsychotics once they commence them, new antipsychotic prescriptions are less commonly initiated than in the past. Over the decade, comparison of both the cross-sectional cohorts, and of the longitudinal, linked cohort, reveal a striking increase in the prescription of antidepressants (11.2% to 19.1%, and 9.9% to 22.0%). This was particularly so for women and for people with mild intellectual disabilities. To a lesser extent, there were also increases in prescribing of hypnotics/anxiolytics and antiepileptics in the linked cohort, but not in the comparison of the cross-sectional cohorts. This difference may be accounted for by the known increase in these prescriptions with age (5), as the linked cohort is of course 10 years older in T2, whereas age and sex are similar in the whole cohorts in T2 and in T1. The age-related change in antiepileptic prescribing in the longitudinal linked cohort, but not in the comparison of the similarly aged cross-sectional cohorts, contextualises the antipsychotic and antidepressant findings (prescribing trends in general), as antiepileptics were almost all prescribed for the highly prevalent condition of epilepsy in this population. Whilst previous studies have reported high rates of antipsychotic prescribing, we are not aware of any that have investigated prescribing over this length of time along with related fluctuations in assessed mental ill-health.

Comparison with previous literature

To our knowledge only two studies have investigated longitudinal psychotropic prescribing patterns in community based samples of people with intellectual disabilities in the UK. Both studies were large and relied upon data extracted from primary care records. One reported antipsychotic prescribing for 17.1%, and antidepressants for 16.9% of adults with intellectual disabilities, with age being associated with both, and sex with antidepressants, similar to our T2 results. (5) The results of the other differed, reporting antipsychotic prescribing in 27.7% of participants at the end of their study period, but also finding a fall of 4% per year over the whole study period, and no consistent trend in antidepressant prescriptions was reported. (5)

For peer review only - http://bmjopen.bmj.com/site/about/guidelines.xhtml

Neither study conducted psychiatric assessments on the population, limiting the precision of findings related to clinical diagnosis and GP recorded symptoms.

This study reaffirms the strong association between antipsychotic prescribing and problem behaviours reported in a number of other studies (28-31). However few studies have separately reported associations between problem behaviours and antidepressants or hynotics/anxiolytics. An Irish study which investigated rates of prescribing of psychotropics in older adults with intellectual disabilities reported no increased risk of antidepressant prescribing or any association with problem behaviours (32).

Several studies have reported the increase in rates of antidepressant prescribing in the general population across the UK, which our findings mirror. (33-35) In Scotland the number of antidepressant prescriptions rose from 1.16 to 3.53 million per year between 1992 and 2006, (36) and women were prescribed antidepressants more frequently than men. (33) The reasons for the increase are unclear and have been attributed to multiple factors such as the availability of newer classes of drugs with fewer side effects, improved management of depression, lack of availability of alternative interventions, (36) a widening of clinical uses (33) and patient expectations. Earlier studies have cited concerns that depression may have been underdiagnosed in the population with intellectual disabilities (37). One American study which retrospectively analysed outpatient psychiatric charts reported a higher than expected rate of antidepressant prescribing for the subgroup with intellectual disabilities and suggested this was indicative of increasing diagnosis of depressive disorders in adults with intellectual disabilities (38). Another US study analysed data from adults with intellectual disabilities living in community settings in New York State between 2006 and 2007 also reported a higher than expected rate of antidressant prescribing in this group (39). The substantial increase in antidepressant prescribing observed in the current study may indicate improved diagnosis in primary care for this population. (24) This study has also observed that problem behaviours were independently associated with antidepressant prescribing in adults with intellectual disabilities. However a systematic review of antidepressants and problem behaviours management in people with intellectual disabilities concluded that evidence of their effectiveness in this context is lacking (40). Longitudinal patterns of antidepressant prescribing require further investigation.

Strengths and limitation

For peer review only - http://bmjopen.bmj.com/site/about/guidelines.xhtml

Strengths of the study include its large size, the longitudinal design, the detailed ascertainment of the population with intellectual disabilities, and the detailed health assessments at T1. The crosssectional cohorts were population-based at T1 and T2, so representative more widely of the population with intellectual disabilities. Additionally, the linked cohort was similar in characteristics with the whole cohort at T1, also suggesting it is representative and hence that the results are generalisable. The period of 12 weeks extraction of PIS data was selected to account for the frequency of prescriptions being issued. It included both regular and as-required drugs; given the 12 week prescribing period it is likely that the as-required medication was being actively used (as a fresh prescription had been issued and was encashed by the person with intellectual disabilities/their carer during this period). The time period for encashment was identical at both time points for the longitudinal, linked cohort. As a matter of caution in interpreting the data, the case-conferenced clinical mental ill-health diagnoses agreed by the study psychiatrists were used rather than ICD-10 or DSM-IV-TR diagnoses, in view of the under-recording of mental ill-health that these two classification systems produce with this population: had we used either of these classifications, our results would have been even more striking in terms of the discrepancy between mental ill-health and prescription of antipsychotics.

Only 73% of general practices agreed to data extraction, and this combined with deaths are likely to be the main reasons for 545/1,190 of the participants being linked in the T2 data, 10 years later. Limitations are the different methods of data collection, with specialist individual assessments at T1 and electronic data extraction at T2; in particular there is a large proportion of missing information and there may be inaccuracies on recorded level of intellectual disabilities in the general practitioner data at T2, so comparison of this variable between the T1 and T2 cohorts is limited. Additionally, there is lack of mental ill-health data at T2. The study did not investigate changes in dosages, polypharmacy, or duration of use. Some antipsychotic drugs are licenced for indications other than psychosis, and it is possible that other conditions accounted for their use e.g. promazine, whereas antidepressants and antiepileptics have seen increased use in the general population over this time period for neuralgic pain. We do not know how relevant this is to people with intellectual disabilities who may have difficulties in communicating pain, and note that encashed antiepileptics did not increase between the two cohorts.

Implications for research and practice

For peer review only - http://bmjopen.bmj.com/site/about/guidelines.xhtml

This study has demonstrated that fewer new antipsychotic prescriptions are being initiated, but those prescribed antipsychotics in T1 were unlikely to have these drugs withdrawn over the next decade demonstrating possible reluctance from carers, families and individuals combined with a lack of evidence available to prescribers to direct cessation interventions (22, 41) . The issue therefore remains far from addressed, and the risks of long-term health problems, death, and impact on quality of life associated with long-term antipsychotic prescriptions still needs further highlighting. (42) This study reinforces the need for frequent medication reviews for people with intellectual disabilities, alongside further research to investigate the long-term effects of antipsychotic medications on this population. (8) Further research to examine the barriers to antipsychotic drug reduction and to evaluate approaches to promoting reduction and withdrawal of antipsychotics for people with intellectual disabilities is needed. There is a dearth of evidence on antidepressant prescribing in the population with intellectual disabilities. The sharp increase in antidepressant prescribing observed in this study demands further research to understand the drivers for this. The association between increasing age and prescribing of antipsychotics and antidepressants also supports calls for research to investigate the implications of long-term psychotropic prescribing on older people with intellectual disabilities.(43)

For peer review only

Competing interests

All authors have completed the ICMJE uniform disclosure form at www.icmje.org/coi_disclosure.pdf and declare: no support from any organisation for the submitted work; no financial relationships with any organisations that might have an interest in the submitted work in the previous three years; no other relationships or activities that could appear to have influenced the submitted work .

Author statement

AH and PMcS analysed the data, jointly interpreted it, AH wrote the first draft of the manuscript, AH, S-AC, DK, CM, and LA jointly conceived the project, interpreted the data and contributed to the manuscript. AMcI contributed to additional analyses in response to reviewer comments. All authors approved the final version of the manuscript. S-AC is the study guarantor.

For peer review only - http://bmjopen.bmj.com/site/about/guidelines.xhtml

Funding

The study was funded by the Greater Glasgow Health Board, the Scottish Government via the Scottish Learning Disabilities Observatory and from the UK Medical Research Council (grant number: MC_PC_1717).

The study sponsors and funders had no role in the study design; in the collection, analysis, and interpretation of data; in the writing of the report; and in the decision to submit the article for publication.

The researchers are independent from the funders.

Statement

All authors had full access to all of the data (including statistical reports and tables) in the study and can take responsibility for the integrity of the data and the accuracy of the data analysis.

Transparency declaration

The manuscript's guarantor (S-AC) affirms that the manuscript is an honest, accurate, and transparent account of the study being reported; that no important aspects of the study have been omitted; and that there were no discrepancies from the study as planned.

Data sharing

No additional data is available

Acknowledgements

We are grateful to all the participants and their carers, to NHS Greater Glasgow learning disabilities primary care liaison team, to NHS Greater Glasgow and Clyde Safe Haven. This work uses data provided by patients and collected by the NHS as part of their care and support.

For peer review only - http://bmjopen.bmj.com/site/about/guidelines.xhtml

REFERENCES

1. Cooper SA, Smiley E, Morrison J, Williamson A, Allan L. Mental ill-health in adults with intellectual disabilities: prevalence and associated factors. Br J Psychiatry. 2007;190:27-35.
2. Cooper SA, Smiley E, Morrison J, Allan L, Williamson A, Finlayson J, et al. Psychosis and adults with intellectual disabilities - Prevalence, incidence, and related factors. Soc Psych Psych Epid. 2007;42(7):530-6.
3. Lunsky Y, Khuu W, Tadrous M, Vigod S, Cobigo V, Gomes T. Antipsychotic Use With and Without Comorbid Psychiatric Diagnosis Among Adults with Intellectual and Developmental Disabilities. Can J Psychiatry. 2017:706743717727240.
4. Lunsky Y, Khuu W, Tadrous M, Vigod S, Cobigo V, Gomes T. Antipsychotic Use With and Without Comorbid Psychiatric Diagnosis Among Adults with Intellectual and Developmental Disabilities. Can J Psychiat. 2018;63(6):361-9.
5. Glover G, Williams R. Prescribing of psychotropic drugs to people with learning disabilities and/or autism by general practitioners in England. London: Public Health England; 2015.
6. Kuijper Gd, Hoekstra P, Visser F, Scholte FA, Penning C, Evenhuis H. Use of antipsychotic drugs in individuals with intellectual disability (ID) in the Netherlands: prevalence and reasons for prescription. Journal of Intellectual Disability Research. 2010;54(7):659-67.
7. NICE. Challenging Behaviour and Learning Disabilities: Prevention and interventions for people with learning disabilities whose behaviour challenge. London: NICE; 2015.
8. Matson JL, Mahan S. Antipsychotic drug side effects for persons with intellectual disability. Res Dev Disabil. 2010;31(6):1570-6.
9. Doan TN, Lennox NG, Taylor-Gomez M, Ware RS. Medication use among Australian adults with intellectual disability in primary healthcare settings: A cross-sectional study. J Intellect Dev Dis. 2013;38(2):177-81.
10. Sheehan R, Hassiotis A, Walters K, Osborn D, Strydom A, Horsfall L. Mental illness, challenging behaviour, and psychotropic drug prescribing in people with intellectual disability: UK population based cohort study. BMJ. 2015;351:h4326.
11. Glassman AH, Bigger JT. Antipsychotic drugs: Prolonged QTc interval, torsade de pointes, and sudden death. Am J Psychiat. 2001;158(11):1774-82.
12. Cornett EM, Novitch M, Kaye AD, Kata V, Kaye AM. Medication-Induced Tardive Dyskinesia: A Review and Update. Ochsner J. 2017;17(2):162-72.
13. Stroup TS, Gray N. Management of common adverse effects of antipsychotic medications. World Psychiatry. 2018;17(3):341-56.
14. Sultan RS, Wang S, Crystal S, Olfson M. Antipsychotic Treatment Among Youths With Attention-Deficit/Hyperactivity Disorder. Jama Netw Open. 2019;2(7).
15. Olfson M, King M, Schoenbaum M. Treatment of Young People With Antipsychotic Medications in the United States. Jama Psychiat. 2015;72(9):867-74.
16. Sultan RS, Correll CU, Schoenbaum M, King M, Walkup JT, Olfson M. National Patterns of Commonly Prescribed Psychotropic Medications to Young People. J Child Adol Psychop. 2018;28(3):158-65.
17. Tyrer P, Cooper SA, Hassiotis A. Drug treatments in people with intellectual disability and challenging behaviour. BMJ. 2014;349:g4323.
18. NICE. Mental health problems in people with learning disabilities: prevention, assessment and management. London: NICE; 2016.
19. Health Do. Transforming care: A national response to Winterbourne View Hospital:

Department of Health Review Final Report. 2012.
20. Disability FoPoI. Psychotropic drug prescribing for people with intellectual disability, mental health problems and/or behaviours that challenge: practice guidelines. London: The Royal College of Psychiatrists; 2016.
21. England N. Stopping over medication of people with a learning disability, autism or both (STOMP) 2019 [29/01/19]. Available from: https://www.england.nhs.uk/learning-disabilities/improving-health/stomp/.

For peer review only - http://bmjopen.bmj.com/site/about/guidelines.xhtml

22. Song M, Ware R, Doan TN, Harley D. Psychotropic medication use in adults with intellectual disability in Queensland, Australia, from 1999 to 2015: a cohort study. J Intellect Disabil Res. 2019.
23. Sparrow SS, Cicchetti DV. Diagnostic Uses of the Vineland Adaptive-Behavior Scales. J Pediatr Psychol. 1985;10(2):215-25.
24. Organization WH. The ICD-10 Classification of Mental and Behavioral Disorders: Diagnostic Criteria for Research. Geneva, Switzerland: World Health Organization; 1993.
25. Association AP. Diagnostic and Statistical Manual of Mental Disorders (4th edn, text revision) (DSM-IV-TR). Washington DC2000.
26. Szymanski LS. DC-LD (Diagnostic criteria for psychiatric disorders for use with adults with learning Disabilities Mental retardation). Journal of Intellectual Disability Research. 2002;46:525-7.
27. Alvarez-Madrazo S, McTaggart S, Nangle C, Nicholson E, Bennie M. Data Resource Profile: The Scottish National Prescribing Information System (PIS). International Journal of Epidemiology. 2016;45(3):714-5f.
28. Sheehan R, Hassiotis A, Walters K, Osborn D, Strydom A, Horsfall L. Mental illness, challenging behaviour, and psychotropic drug prescribing in people with intellectual disability: UK population based cohort study. Bmj-Brit Med J. 2015;351.
29. O'Dwyer C, McCallion P, Henman M, McCarron M, O'Leary E, Burke E, et al. Prevalence and patterns of antipsychotic use and their associations with mental health and problem behaviours among older adults with intellectual disabilities. J Appl Res Intellect Disabil. 2019;32(4):981-93.
30. Deb S, Fraser W. The Use of Psychotropic Medication in People with Learning-Disability - Towards Rational Prescribing. Hum Psychopharm Clin. 1994;9(4):259-72.
31. Bowring DL, Totsika V, Hastings RP, Toogood S, McMahon M. Prevalence of psychotropic medication use and association with challenging behaviour in adults with an intellectual disability. A total population study. J Intellect Disabil Res. 2017;61(6):604-17.
32. Axmon A, El Mrayyan N, Eberhard J, Ahlstrom G. Pharmacotherapy for mood and anxiety disorders in older people with intellectual disability in comparison with the general population. BMC Psychiatry. 2019;19(1):238.
33. Lockhart P, Guthrie B. Trends in primary care antidepressant prescribing 1995-2007: a longitudinal population database analysis. Br J Gen Pract. 2011;61(590):e565-72.
34. Munoz-Arroyo R, Sutton M, Morrison J. Exploring potential explanations for the increase in antidepressant prescribing in Scotland using secondary analyses of routine data. Br J Gen Pract. 2006;56(527):423-8.
35. Hollinghurst S, Kessler D, Peters TJ, Gunnell D. Opportunity cost of antidepressant prescribing in England: analysis of routine data. Brit Med J. 2005;330(7498):999-1000.
36. Morrison J, Anderson MJ, Sutton M, Munoz-Arroyo R, McDonald S, Maxwell M, et al. Factors influencing variation in prescribing of antidepressants by general practices in Scotland. Br J Gen Pract. 2009;59(559):e25-31.
37. Robertson J, Emerson E, Gregory N, Hatton C, Kessissoglou S, Hallam A. Receipt of psychotropic medication by people with intellectual disability in residential settings. Journal of Intellectual Disability Research. 2000;44:666-76.
38. Hurley AD, Folstein M, Lam N. Patients with and without intellectual disability seeking outpatient psychiatric services: diagnoses and prescribing pattern. J Intellect Disabil Res. 2003;47(Pt 1):39-50.
39. Tsiouris JA, Kim SY, Brown WT, Pettinger J, Cohen IL. Prevalence of Psychotropic Drug Use in Adults with Intellectual Disability: Positive and Negative Findings from a Large Scale Study. Journal of Autism and Developmental Disorders. 2013;43(3):719-31.
40. Sohanpal SK, Deb S, Thomas C, Soni R, Lenotre L, Unwin G. The effectiveness of antidepressant medication in the management of behaviour problems in adults with intellectual disabilities: a systematic review. J Intellect Disabil Res. 2007;51(Pt 10):750-65.
41. McNamara R, Randell E, Gillespie D, Wood F, Felce D, Romeo R, et al. A pilot randomised controlled trial of community-led ANtipsychotic Drug REduction for Adults with Learning Disabilities. Health Technol Assess. 2017;21(47):1-92.
42. Scheifes A, Walraven S, Stolker JJ, Nijman HL, Egberts TC, Heerdink ER. Adverse events and the relation with quality of life in adults with intellectual disability and challenging behaviour using psychotropic drugs. Res Dev Disabil. 2016;49-50:13-21.

For peer review only - http://bmjopen.bmj.com/site/about/guidelines.xhtml

43. O'Dwyer M, Peklar J, Mulryan N, McCallion P, McCarron M, Henman MC. Prevalence, patterns and factors associated with psychotropic use in older adults with intellectual disabilities in Ireland. Journal of Intellectual Disability Research. 2017;61(10):969-83.

Figure legends

Figure 1. Participant flow diagram

Figure 2. Types of mental ill-health experienced by people prescribed antipsychotics at T1 n=292

For peer review only

Figure 1. Participant flow diagram

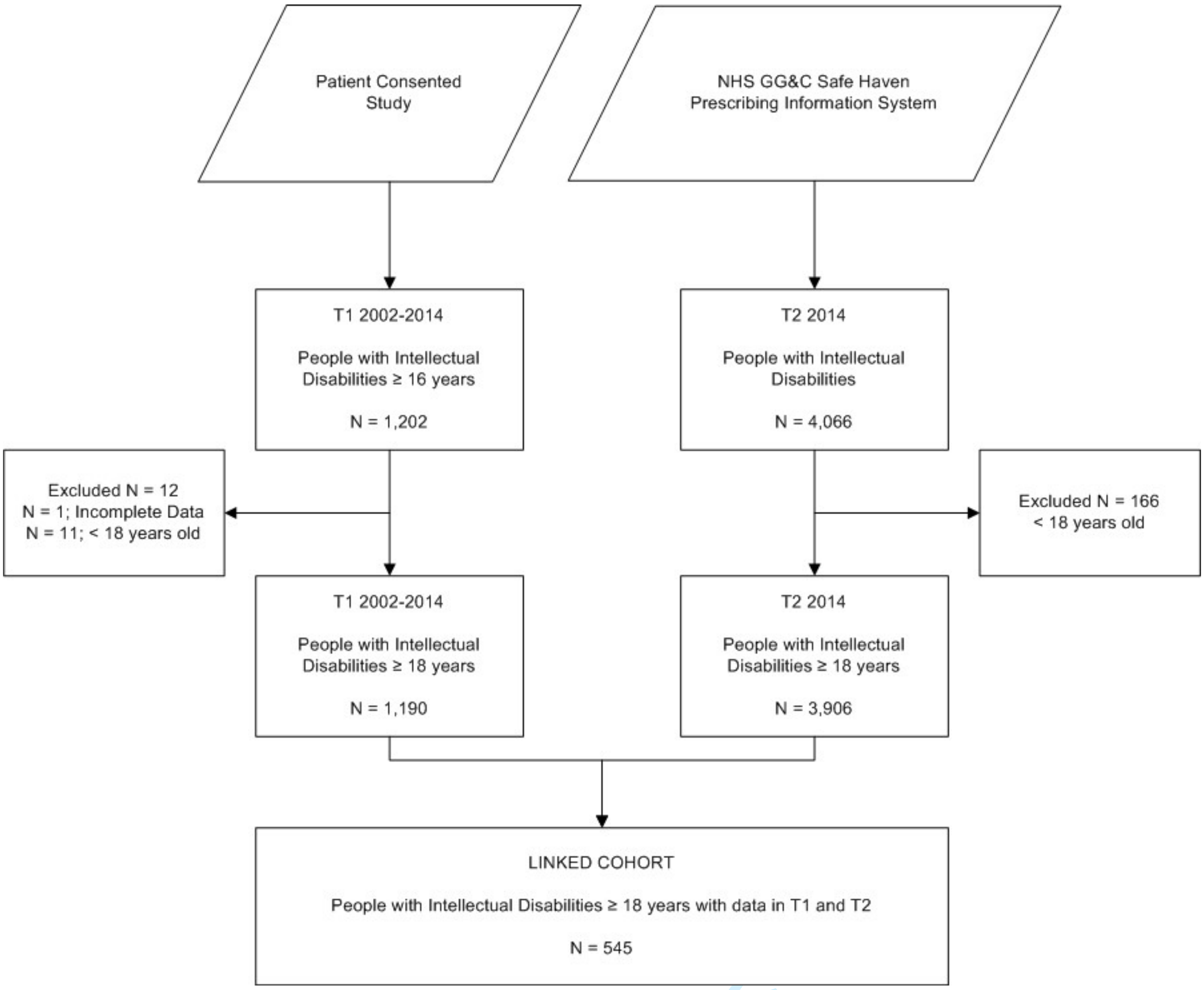

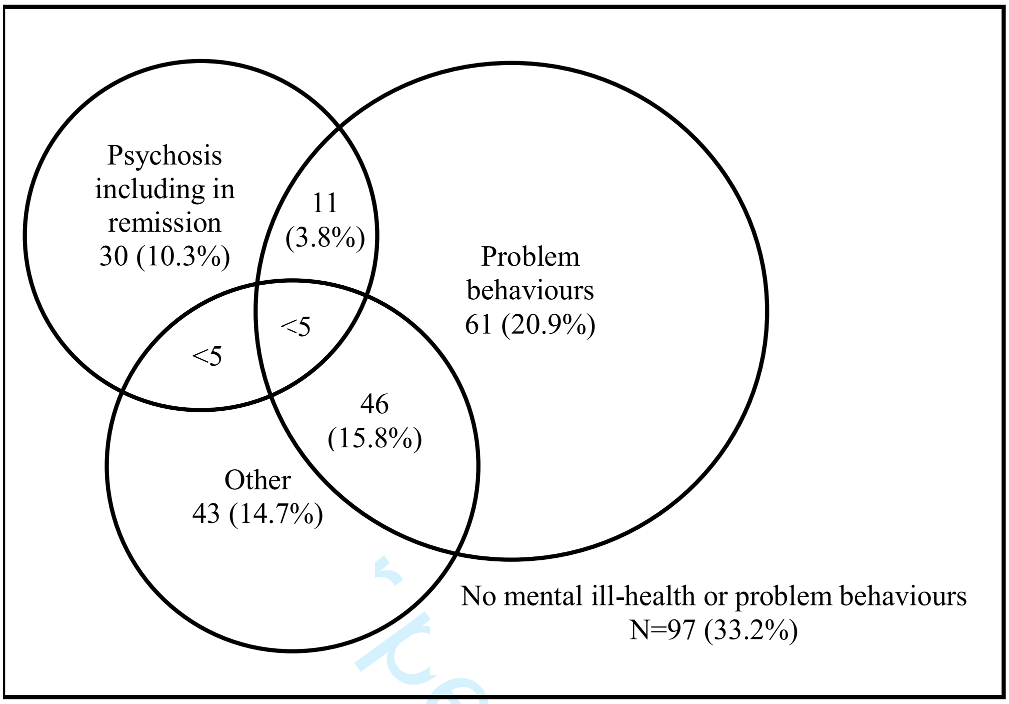

Figure 2. Types of mental ill-health experienced by people prescribed antipsychotics at T1 n=292

For peer review only

Supplementary table 1: Multivariable analysis of factors at T1 associated with psychotropic prescriptions at T2 for the linked cohort (N=545)

	Antipsychotics		Antidepressants		Hypnotics/anxiolytics		Antiepileptics		Lithium*	
	OR (95% CI)	p-value	OR (95% CI)	p-value	OR (95% CI)	p-value	OR (95% CI)	p-value	OR (95% CI)	p-value
Male sex	0.96 (0.63, 1.47)	0.848	0.59 (0.38, 0.89)	0.013	0.83 (0.46, 1.50)	0.545	0.97 (0.66, 1.42)	0.874	0.92 (0.25, 1.09)	0.894
Age at T1	1.04 (1.02, 1.06)	<0.001	1.01 (1.00, 1.03)	0.167	1.00 (0.98, 1.02)	0.993	0.99 (0.98, 1.00)	0.158	1.04 (0.99, 1.09)	0.160
Level of ID	-	0.018	-	0.002	-	0.473	-	< 0.001	-	0.464
Mild	REF		REF		REF		REF		REF	
Moderate	1.71 (1.02, 2.87)	0.041	0.90 (0.55, 1.48)	0.680	1.16 (0.56, 2.38)	0.690	1.56 (0.99, 2.48)	0.057	2.91 (0.68, 12.53)	0.152
Severe	2.31 (1.29, 4.15)	0.005	0.57 (0.30, 1.08)	0.083	0.75 (0.28, 1.97)	0.554	1.66 (0.96, 2.86)	0.071	0.90 (0.09, 8.80)	0.928
Profound	1.07 (0.53, 2.18)	0.853	0.22 (0.09, 0.56)	0.001	1.71 (0.71, 4.11)	0.229	3.57 (1.96, 6.5)	0.001	1.39 (0.14, 13.74)	0.780
Mental ill-health**	3.50 (2.02, 6.06)	< 0.001	2.73 (1.58, 4.70)	< 0.001	2.79 (1.34, 5.81)	0.006	1.33 (0.78, 2.27)	0.294	-	-
Problem behaviours	5.47 (3.25, 9.22)	< 0.001	2.75 (1.59, 4.76)	< 0.001	2.08 (1.00, 4.34)	0.050	1.35 (0.83, 2.17)	0.225	-	-

*Mental illness and problem behaviours excluded from Lithium model due to small numbers **Not including problem behaviours

For peer review only

STROBE (Strengthening The Reporting of OBservational Studies in Epidemiology) Checklist

A checklist of items that should be included in reports of observational studies. You must report the page number in your manuscript where you consider each of the items listed in this checklist. If you have not included this information, either revise your manuscript accordingly before submitting or note N/A.

Note: An Explanation and Elaboration article discusses each checklist item and gives methodological background and published examples of transparent reporting. The STROBE checklist is best used in conjunction with this article (freely available on the Web sites of PLoS Medicine at http://www.plosmedicine.org/, Annals of Internal Medicine at http://www.annals.org/, and Epidemiology at http://www.epidem.com/). Information on the STROBE Initiative is available at www.strobe-statement.org.

Section and Item	Item No.	Recommendation	Reported on Page No.
**Title and Abstract**	1	(*a*) Indicate the study's design with a commonly used term in the title or the abstract	
		(*b*) Provide in the abstract an informative and balanced summary of what was done and what was found	
**Introduction**			
Background/Rationale	2	Explain the scientific background and rationale for the investigation being reported	
Objectives	3	State specific objectives, including any prespecified hypotheses	
**Methods**			
Study Design	4	Present key elements of study design early in the paper	
Setting	5	Describe the setting, locations, and relevant dates, including periods of recruitment, exposure, follow-up, and data collection	
Participants	6	(*a*) *Cohort study*—Give the eligibility criteria, and the sources and methods of selection of participants. Describe methods of follow-up<br>*Case-control study*—Give the eligibility criteria, and the sources and methods of case ascertainment and control selection. Give the rationale for the choice of cases and controls<br>*Cross-sectional study*—Give the eligibility criteria, and the sources and methods of selection of participants	
		(*b*) *Cohort study*—For matched studies, give matching criteria and number of exposed and unexposed<br>*Case-control study*—For matched studies, give matching criteria and the number of controls per case	
Variables	7	Clearly define all outcomes, exposures, predictors, potential confounders, and effect modifiers. Give diagnostic criteria, if applicable	

For peer review only

Section and Item	Item No.	Recommendation	Reported on Page No.
Data Sources/ Measurement	8*	For each variable of interest, give sources of data and details of methods of assessment (measurement). Describe comparability of assessment methods if there is more than one group	
Bias	9	Describe any efforts to address potential sources of bias	
Study Size	10	Explain how the study size was arrived at	
Quantitative Variables	11	Explain how quantitative variables were handled in the analyses. If applicable, describe which groupings were chosen and why	
Statistical Methods	12	(*a*) Describe all statistical methods, including those used to control for confounding	
		(*b*) Describe any methods used to examine subgroups and interactions	
		(*c*) Explain how missing data were addressed	
		(*d*) *Cohort study*—If applicable, explain how loss to follow-up was addressed<br>*Case-control study*—If applicable, explain how matching of cases and controls was addressed<br>*Cross-sectional study*—If applicable, describe analytical methods taking account of sampling strategy	
		(*e*) Describe any sensitivity analyses	
**Results**			
Participants	13*	(a) Report numbers of individuals at each stage of study—eg numbers potentially eligible, examined for eligibility, confirmed eligible, included in the study, completing follow-up, and analysed	
		(b) Give reasons for non-participation at each stage	
		(c) Consider use of a flow diagram	
Descriptive Data	14*	(a) Give characteristics of study participants (eg demographic, clinical, social) and information on exposures and potential confounders	
		(b) Indicate number of participants with missing data for each variable of interest	
		(c) *Cohort study*—Summarise follow-up time (eg, average and total amount)	
Outcome Data	15*	*Cohort study*—Report numbers of outcome events or summary measures over time	
		Case-control study—Report numbers in each exposure category, or summary measures of exposure	
		Cross-sectional study—Report numbers of outcome events or summary measures	

For peer review only

Section and Item	Item No.	Recommendation	Reported on Page No.
Main Results	16	(*a*) Give unadjusted estimates and, if applicable, confounder-adjusted estimates and their precision (eg, 95% confidence interval). Make clear which confounders were adjusted for and why they were included	
		(*b*) Report category boundaries when continuous variables were categorized	
		(*c*) If relevant, consider translating estimates of relative risk into absolute risk for a meaningful time period	
Other Analyses	17	Report other analyses done—eg analyses of subgroups and interactions, and sensitivity analyses	
**Discussion**			
Key Results	18	Summarise key results with reference to study objectives	
Limitations	19	Discuss limitations of the study, taking into account sources of potential bias or imprecision. Discuss both direction and magnitude of any potential bias	
Interpretation	20	Give a cautious overall interpretation of results considering objectives, limitations, multiplicity of analyses, results from similar studies, and other relevant evidence	
Generalisability	21	Discuss the generalisability (external validity) of the study results	
**Other Information**			
Funding	22	Give the source of funding and the role of the funders for the present study and, if applicable, for the original study on which the present article is based	

*Give information separately for cases and controls in case-control studies and, if applicable, for exposed and unexposed groups in cohort and cross-sectional studies.

Once you have completed this checklist, please save a copy and upload it as part of your submission. DO NOT include this checklist as part of the main manuscript document. It must be uploaded as a separate file.

For peer review only
